# Subphthalocyanine Platform for Single-Molecule Machines on Surface: Ligand-Directed Adsorption on Au(111)

**DOI:** 10.1021/acsnano.5c17283

**Published:** 2026-03-12

**Authors:** Franz Plate, Soyoung Park, Ebru Cihan, Natasha Khera, Ningwei Sun, Pranjit Das, Olga Guskova, Dmitry A. Ryndyk, Franziska Lissel, Francesca Moresco

**Affiliations:** † Center for Advancing Electronics Dresden, 9169 TU Dresden, 01062 Dresden, Germany; ‡ 28408 Leibniz Institute of Polymer Research Dresden, Hohe Strasse 6, 01069 Dresden, Germany; § Institute for Applied Polymer Physics, TU Hamburg, 21073 Hamburg, Germany

**Keywords:** subphthalocyanine, axial ligands, Au(111), scanning tunneling microscopy, molecular simulation

## Abstract

For the development of single-molecule machines on surfaces, a vertical molecular geometry based on a simple, common platform is a promising design approach. This could allow decoupling of the active unit from the supporting surface and obtaining of a flexible modular system. An ideal platform for this purpose is subphthalocyanine with its bowl-shaped geometry and axial functionalization. We functionalized SubPcs with a series of vertical, axial ligands with varying conjugation lengths. Their adsorption on the Au(111) surface was studied by low-temperature scanning tunneling microscopy, supported by simulations. We found that increasing the conjugation length of the axial ligand induces a distinct transition in the adsorption geometry. Long ligands, such as azobenzene and naphthalene derivatives, adopt a reverse adsorption geometry with the ligand adsorbed flat on the surface and the SubPc platform pointing upward. These reverse molecules further interact, forming one-dimensional chains. The intermolecular arrangement and distances in the chains are determined by the orientation of the axial ligand on the surface. In contrast, the shortest ligand, which is formed by a single phenyl ring derivative, predominantly adsorbs with the SubPc platform on the surface and allows rotation by the STM tip. Our findings reveal a clear structure–adsorption relationship and offer a rational strategy to control the orientation and packing of SubPc-based single-molecule machines on surfaces through the design of the axial ligands.

## Graphical abstract

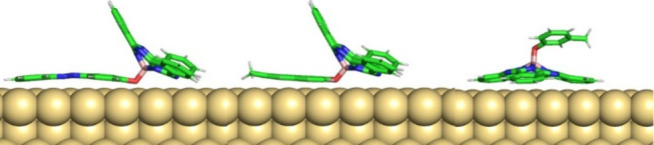

The rational design of flexible, modular molecular systems is presently in the focus of research for the development of single-molecule machines that can perform calculations, produce work, and store energy.^1^ Molecular systems based on a common platform and variable functional axial ligands could allow the exploration of comparable structures, contributing to our understanding of how electronic excitations transform into motion at the nanoscale. Designing the platform and the active functional units separately may allow for various combinations of active units on different platforms, enabling controllable tuning of the nanomachine properties. Furthermore, a stable platform should enable a vertical adsorption geometry with the active unit isolated from the metal surface, thus allowing the storage of energy for a finite, long time.

A promising flexible system that we propose to use as a platform for single-molecule machines is subphthalocyanine (SubPc).^2^ SubPc is a 3-fold symmetric aromatic macrocycle composed of three *N*-fused 1,3-diiminoisoindole units arranged around a boron atom, which also bears an axial substituent. Simple SubPc molecules equipped with a Cl axial ligand (SubPc-Cl) were studied by STM on Cu, Ag, and Au surfaces, showing the formation of ordered self-assembled structures that respect both the symmetry of the molecule and the geometry of the substrate.^3–8^ Interestingly, the center axial coordination site of SubPc points away from the plane of the molecule and can be used for the attachment of vertically oriented axial ligand molecules,^3,4^ thus offering a possible versatile synthetic platform for single-molecule machines. In this context, we investigated the adsorption properties of SubPc functionalized with axial ligands of different lengths to optimize the dimensions of the active unit for a working single-molecule machine.

A few examples of vertical rotors based on a stator platform have been presented in the last years, including the trioxatriangulenium (TOTA) platform,^9^ and organometallic double-decker rotors where the platform is connected to the rotating or switching unit by a metal atom.^10,11^ Single-molecule machines based on *N*-heterocyclic carbene (NHC) platforms have also been studied, showing a variety of coupling and adsorption geometries.^12–15^

Here we present the synthesis and adsorption of three different molecules based on the same SubPc platform. The axial ligands were designed to have different conjugation lengths. By low-temperature STM (LT-STM) supported by density functional theory (DFT) and molecular dynamics (MD) simulations, we show that the adsorption geometry of the molecules depends critically on the extension of the ligand. SubPc equipped with long ligands, such as phenylazophenoxy and 6-methylnaphthoxy (**SubPc-Azo** and **SubPc-MN**, respectively), present a reverse adsorption geometry, with the ligand adsorbed on the surface and the SubPc platform oriented nearly vertically. At submonolayer coverage, the reverse-adsorbed molecules assemble into apparently similar one-dimensional chains. The orientations and distances of the molecules in these chains are governed by the axial ligand on the surface. By further reducing the length of the ligand, we observe that SubPc-3-methylphenoxy (**SubPc-MP**) adsorbs in most cases vertically, with the SubPc platform parallel to the surface, and is arranged similarly to plane SubPc. By lateral manipulation with the STM tip, the **MP** ligand can be controllably rotated.

## Results and Discussion

### Design and Synthesis

Axially halogenated SubPcs, such as boron subphthalocyanine chloride (SubPc–Cl), serve as versatile synthetic platforms due to the high reactivity of the axial boron–halogen bond.^16,17^ This reactivity allows efficient axial ligand substitution under standard reflux conditions, enabling structural variability without disrupting the SubPc platform.^16,17^ In this study, three molecules, **SubPc-Azo** (4-phenylazophenoxyboronsubphthalocyanine), **SubPc-MN** (6-methylnaphthoxyboronsubphthalocyanine), and **SubPc-MP** (3-methylphenoxyboronsubphthalocyanine), were synthesized by substituting the axial chlorine ligand of boron subphthalocyanine chloride with three different units: phenylazophenoxy (4-hydroxyazobenzene), 6-methylnaphthoxy and 3-methylphenoxy groups (Scheme 1). These substituents were selected to explore how the length and π-extension of the axial ligand influence the adsorption geometry and molecular assembly on surfaces. The synthesis followed a general procedure adapted from previously reported methods^18,19^ and is described in the Supporting Information (SI) file.

**Scheme 1:**
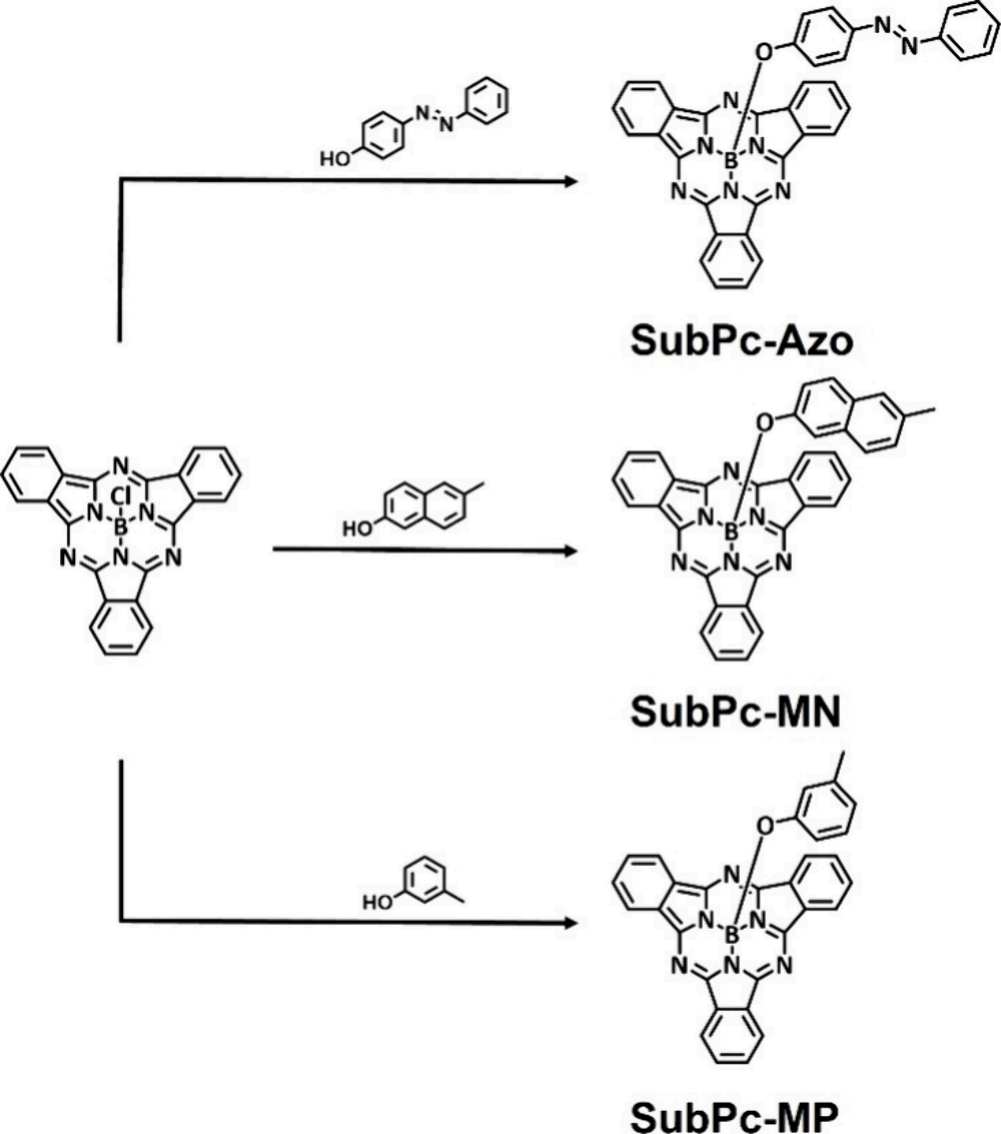
Synthesis of Three Different Axial Ligands on a SubPc Platform: Long Ligand **SubPc-Azo**; Middle Ligand **SubPc-MN**; Short Ligand **SubPc-MP**

### Reverse Adsorption of SubPc-Azo and SubPc-MN

We first investigated by LT-STM the adsorption of the two SubPcs equipped with longer axial ligands: **SubPc-Azo** and **SubPc-MN** (Figure 1) on the Au(111) surface.

**Figure 1:**
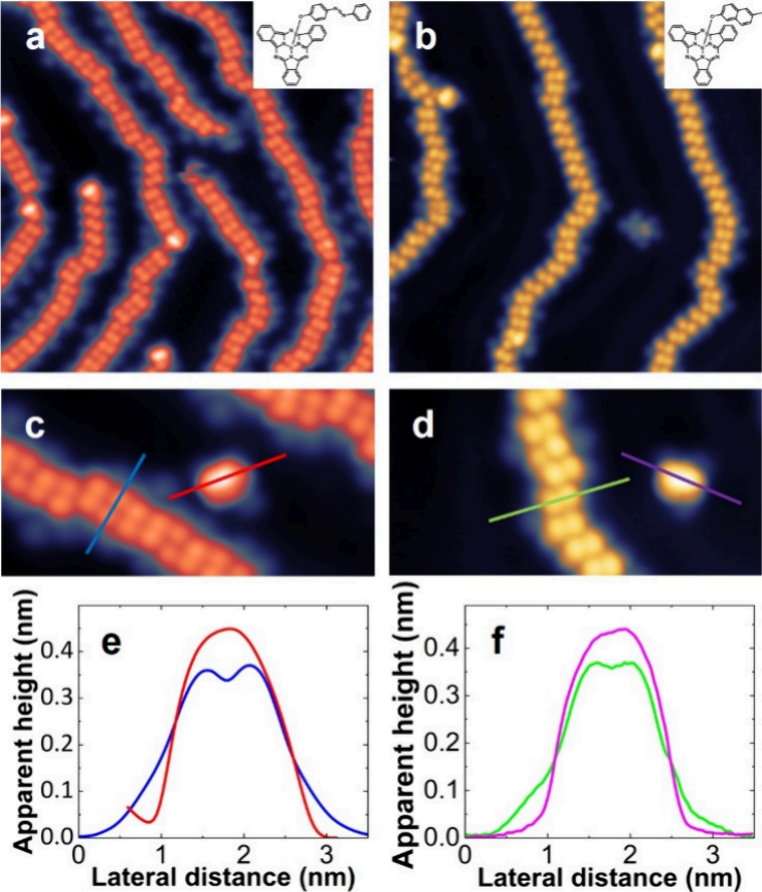
STM images of **SubPc-Azo** and **SubPc-MN** on Au(111). (a) Overview image of **SubPc-Azo** (image parameters: *U* = 0.5 V, *I* = 20 pA, size 20 nm × 20 nm); (b) overview image of **SubPc-MN** (*U* = 0.2 V, *I* = 20 pA, size 20 nm × 20 nm), the molecular structures are shown in the insets of (a) and (b); (c) single **SubPc-Azo** close to a 1D chain (*U* = 0.5 V, *I* = 10 pA, size 10 nm × 5 nm); (d) single **SubPc-MN** close to a 1D chain (*U* = 0.2 V, *I* = 8 pA, size 10 nm × 5 nm); (e) comparison of the linescans across the chain and along the isolated molecule for **SubPc-Azo**, taken along the blue and red lines in (c); and (f) comparison of the linescans across the chain and along the isolated molecule for **SubPc-MN**, taken along the green and violet lines in (d).

After deposition at submonolayer coverage, we observe in the STM images (Figure 1a,b) that both molecules form long and similar one-dimensional (1D) chains following the Au(111) reconstruction (see also SI Figure S17). In Figure 1c,d, we compare the chains with single isolated molecules for both ligands. In both cases, the molecules show a different conformation when adsorbed at the end of the chain or when isolated from the chain. As visible also in the linescans of Figure 1e,f, the isolated molecules show one single higher lobe, while the chains are formed by rows of smaller parallel lobes. Interestingly, if we now compare **SubPc-Azo** with **SubPc-MN** (i.e., Figure 1c,e with d,f), we notice not only a strong similarity of the chains but also that the isolated molecules appear almost identical in the STM images and linescans, which is surprising considering the different conjugation lengths of the axial ligands (see Scheme 1). Furthermore, the formation of chains and the appearance of the molecules do not resemble the known appearance and assemblies of the SubPc base, which is known to show a triangular shape and to self-assemble into 2D structures on Au(111).^12,13^

To isolate a single molecule (Figure 1c,d), we have used STM tip manipulation, as shown in the example of Figure 2 for **SubPc-Azo**. By lateral manipulation (Figure 2a), we open the chain and extract a molecule, which appears as a single higher lobe (Figure 2a, lower panel). In Figure 2b, we have manipulated the same **SubPc-Azo** back to the chain by applying a voltage pulse, thus reconstructing the 1D chain with its typical parallel aligned smaller lobes (see Movie M1 and the complete sequence in the SI Figure S16). Further manipulation experiments for both axial ligands (see SI Figures S18–S21) confirm that the one-lobe conformation is stable for isolated molecules, while the two-lobe conformation seems to be stabilized by the presence of two neighbor molecules of the chain.

**Figure 2:**
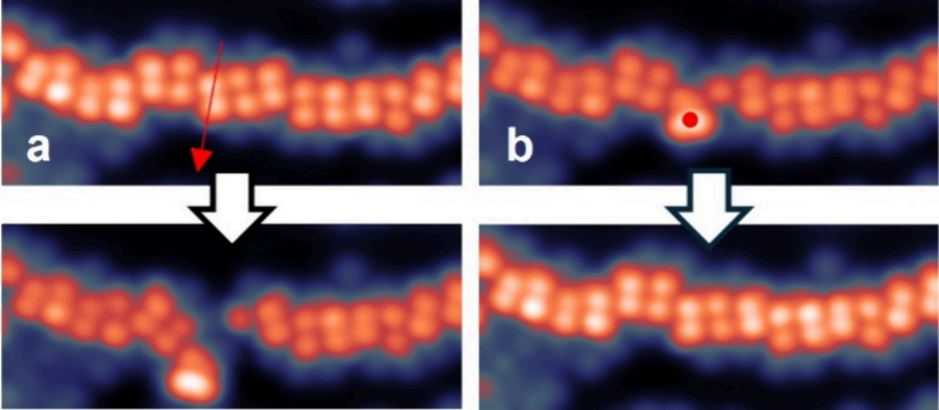
Example of manipulation of a **SubPc-Azo** 1D chain. (a) Lateral manipulation, (*V* = 0.01 V, *I* = 0.3 nA) moving the STM tip along the red arrow; and (b) voltage pulse manipulation (*V* = 3 V, constant height mode) at the position of the red dot. Upper and lower panels show the STM images before and after the respective manipulation. Images parameters: *U* = 0.5 V, *I* = 10 pA, size 10 nm × 4 nm. The complete sequence is shown in SI Figure S16 and Movie M1.

To understand the observed adsorption geometries and the chain formation for the two different axial ligands, we performed classical force field molecular dynamics (MD), quantum DFT calculations, and STM image simulations (see Methods for details). Figure 3 presents the simulation results for a single **SubPc-Azo** molecule adsorbed on Au(111). A similar case of **SubPc-MN** is shown in the SI Figure S30. In both cases, when a molecule lands on the Au(111) surface, it first interacts with the ligand unit (i.e., phenylazophenoxy or 6-methylnaphthoxy). Due to its long conjugation length, the ligand adsorbs flat while the SubPc base remains nearly vertical (see Supplementary Movie M2 for the adsorption dynamics). Two possible stable reverse adsorption geometries result: one with two phenyl rings of the SubPc unit pointing out of the surface (Figure 3a–c) and the other with two phenyl rings adsorbed on the surface and the third pointing out (Figure 3d–f).

**Figure 3:**
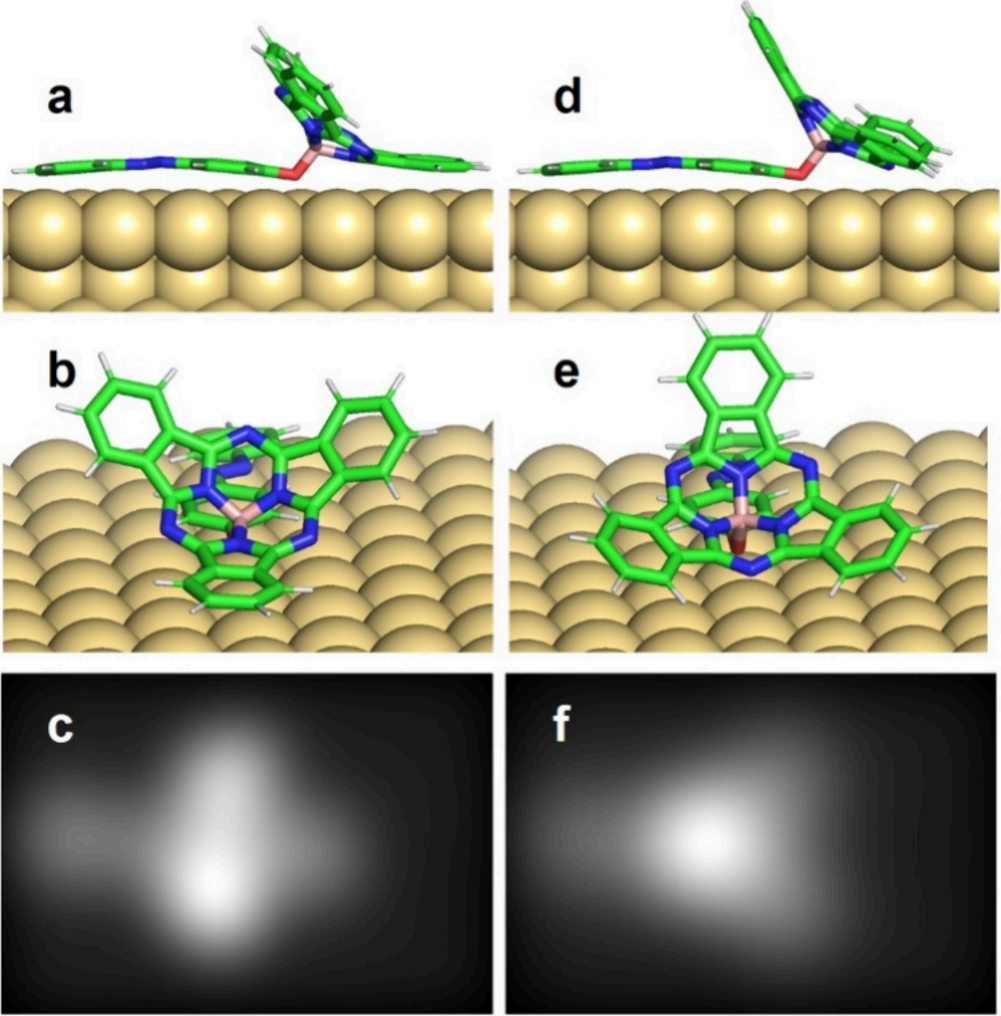
Reverse adsorption geometry of **SubPc-Azo** calculated by DFT. (a) Conformation with two phenyl rings of the SubPc unit pointing out of the surface (two-lobe conformation); (b) corresponding view on the SubPc base; (c) corresponding simulated STM images (image size 3.0 nm × 2.0 nm); (d) conformation with two phenyl rings adsorbed on the surface and the third pointing out (one-lobe conformation); (e) corresponding view on the SubPc base; and (f) corresponding simulated STM images (image size 3.0 nm × 2.0 nm). The two similar conformations for **SubPc-MN** are reported in SI Figure S30.

Our DFT calculations show that the energy of the conformation with two phenyl rings pointing down (Figure 3d–f) is about 0.1 eV lower than the other. This indicates that already at a temperature of 5 K this conformation becomes favorable.

By comparing the simulated results of Figure 3 with those of Figure 1, we can assign the reverse conformation with only one phenyl ring pointing out (Figure 3d–f) to the isolated molecules with a single higher lobe (“one-lobe” conformation). The double lobe in the chains, on the other hand, clearly resembles the conformation with two phenyl groups vertically oriented as in Figure 3a–c (“two-lobes” conformation).

Such interpretation also nicely explains the manipulation sequence of Figure 2 (see also SI Movie M1 and Figure S16), where a **SubPc-Azo** of the chain (in the two-lobe conformation as in Figure 3a–c) can be extracted by manipulation, changing its conformation by the rotation of SubPc to the one-lobe conformation with one phenyl group pointing out (as in Figure 3d–f). The same molecule (Figure 2b) can be reinserted in the chain, rotating back to two phenyl groups pointing up. The rotation of the vertically oriented SubPc resembles the rotation of the SubPc-double-wheel molecule that we demonstrated in refs 20, 21 . However, the exact orientation of the reverse-adsorbed molecules in the chains critically depends on how the molecules diffuse on the surface and interact with each other during the chain formation.

### Chain Formation and Geometry for SubPc-Azo and SubPc-MN

When comparing the STM images of **SubPc-Azo** and **SubPc-MN** in Figure 1, we observe that despite having different ligands, both functionalized SubPcs appear almost identical when isolated but also the chains are very similar due to the reverse adsorption discussed above. However, there are small differences in the lobe–lobe distance and in the stacking of the molecules, as also visible in the STM images and linescans of Figure S29.

To understand the chain formation and the molecule–molecule interaction, we have performed MD simulations, describing the dynamics of the chain formation. The simulation of small ensembles of four **SubPc-Azo** and **SubPc-MN** on the surface shows the formation of 1D chains with different structure (Supplementary Movies M3, M4, M5, M6). DFT calculations confirm these results. The simulation results are compared with those from the experiment in Figure 4.

**Figure 4:**
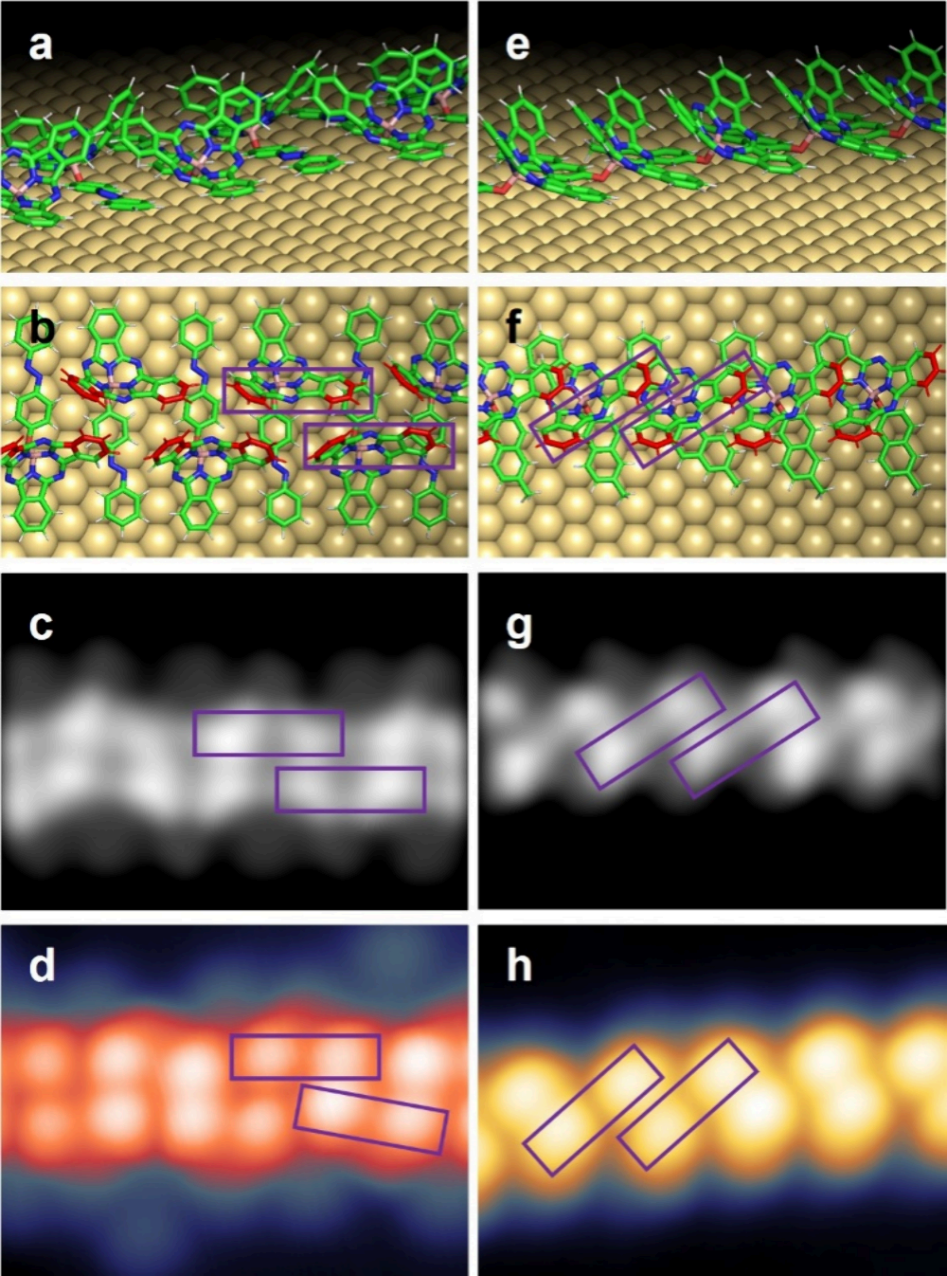
Chain formations for **SubPc-Azo** and **SubPc-MN**. (a) Calculated adsorption geometry of **SubPc-Azo** in side view; (b) corresponding top view, in red we marked the top segments of the phenyl rings responsible for the maxima in the STM images; (c) simulated STM image of a **SubPc-Azo** chain, *U* = 0.5 V, size 4 nm × 3 nm; (d) experimental STM image of a **SubPc-Azo** chain (*U* = 0.5 V, *I* = 2.0 pA, size 4 nm × 3 nm); (e) DFT calculation of the adsorption geometry of **SubPc-MN** in side view: (f) corresponding top view, in red we marked the top segments of the phenyl rings responsible for the maxima in the STM images; (g) simulated STM image of a **SubPc-MN** chain, *U* = 0.5 V, size 4 nm × 3 nm; and (h) experimental STM image of a **SubPc-MN** chain (*U* = 0.5 V, *I* = 2.0 pA, size 4 nm × 3 nm). The rectangles superposed on the figures are a guide to the eye and indicate the positions of the SubPc groups in the chains.

We consider **SubPc-Azo** (Figure 4a–d). DFT calculations of the adsorption geometry of the **SubPc-Azo** chains are shown in Figure 4a,b and indicate that the extended phenylazophenoxy ligand induces an alternating stacking of the SubPc-group facing each other. The axial ligands are oriented away from the chain of the interacting SubPc groups. The corresponding simulated STM images in Figure 4c are in very good agreement with the experimental image in Figure 4d (see also the linescan comparison in the SI Figure S31).

On the other hand, the **SubPc-MN** molecules mainly form a parallel symmetric stacking, slightly shifted on Au(111) due to the functionalization with a shorter axial ligand (Figure 4e,f). The 6-methylnaphthoxy groups are oriented next to each other, forcing a slight shift in the SubPc groups facing up. Also in this case, the very good agreement between the simulated STM image (Figure 4g) and the experimental image (Figure 4h) confirms the adsorption geometry, indicating the importance of the ligands for ordering on the surface. As one can notice in Figure 4b,f, the naphthalene and azobenzene ligands are flexible and can assume different orientations on the surface relative to the core molecule.

In addition, we performed a co-deposition of **SubPc-MN** and pure boron subphthalocyanine chloride (**Cl-SubPc**) to investigate the influence of different adjacent molecules on the adsorption behavior. No different ordering could be achieved in the presence of the flat-adsorbed SubPc and the two molecules form independent structures (Figure S23).

In all cases where 1D chains form, the molecules within the chain are in a two-lobe conformation, with two clearly visible lobes in the STM image. Our force field and DFT calculations show that the intermolecular interaction in the 1D chains, calculated by removing one molecule from the chain, is in the order of 0.7 eV for **SubPc-MN** and 1 eV for **SubPc-Azo**. This value significantly exceeds the difference between the one- and two-lobe conformations (0.1 eV) and explains the preferential formation of one-dimensional chains at sufficiently high substrate temperatures during deposition. On the other hand, the energy changes between different 1D conformations (e.g., shifts between neighbor molecules perpendicular to the chain direction) require smaller energies, explaining different orderings of the 1D chains.

### Adsorption of SubPc-MP

Finally, we considered the third SubPc, functionalized with a shorter 3-methylphenoxy (Scheme 1, overview STM image in Figure S24) that shows a different adsorption geometry and appearance compared with **SubPc-Azo** and **SubPc-MN**.

At submonolayer coverage, we observe molecules forming hexagons, dimers, trimers, and pentamers (Figure 5), while single molecules can rarely be found and are highly mobile. If we compare the observed structures with the previously reported self-assembly of plane SubPc on Au(111),^13^ we find a very good agreement for hexagons and dimers (Figure 5a,b), indicating a direct planar adsorption of the SubPc on the surface. In a few cases, **SubPc-MP** assemblies show one central molecule that appears more intense in the STM images (Figure 5c).

**Figure 5:**
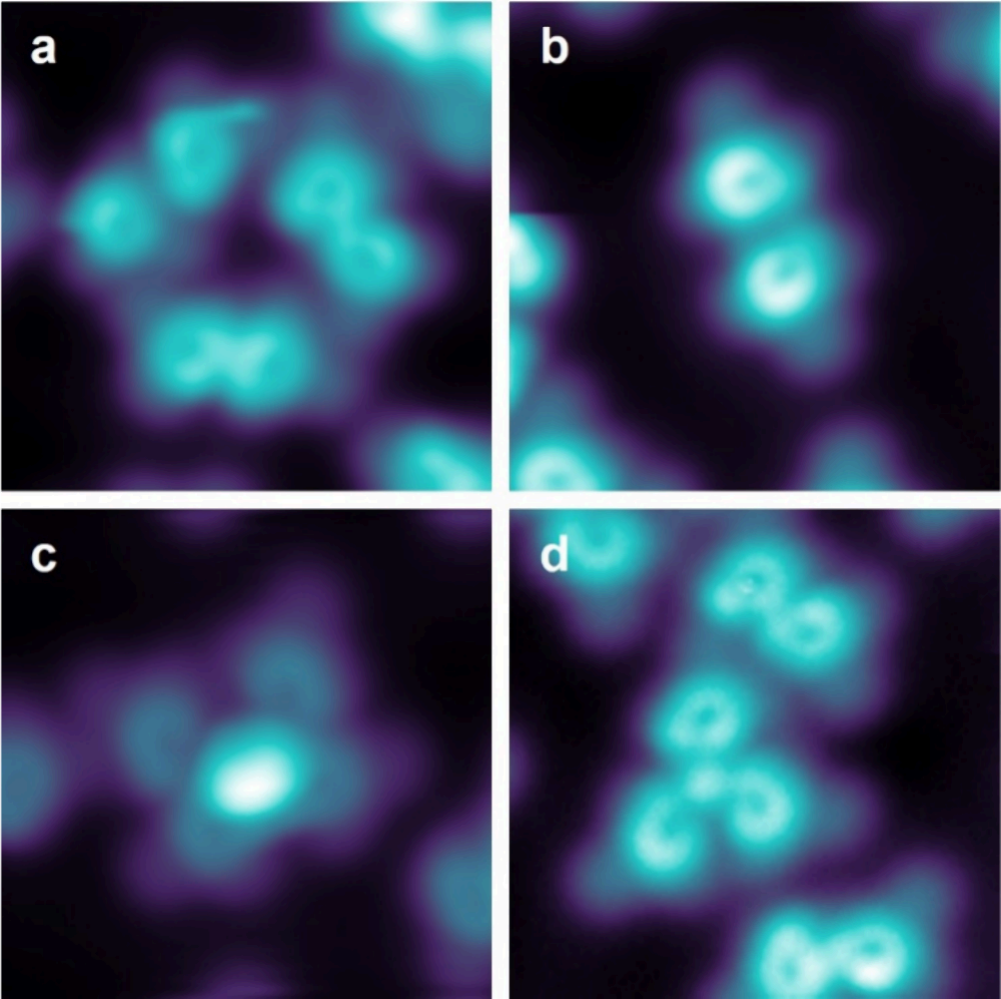
STM image of **SubPc-MP** on Au(111) showing different adsorption assemblies and geometries. (a) Hexagonal structure (*U* = 0.5 V, *I* = 9 pA, size 5 nm × 5 nm); (b) dimer (*U* = 0.5 V, *I* = 9 pA, size 5 nm × 5 nm); (c) pentamer with a reverse-adsorbed molecule in the center (*U* = 0.2 V, *I* = 1 pA, size 5 nm × 5 nm); and (d) trimer (*U* = 0.5 V, *I* = 9 pA, size: 5 nm × 5 nm).

By lateral manipulation, we can separate it from the surrounding molecules (SI Figure S25 and Movie M7), obtaining an isolated **SubPc-MP** with a different conformation (that we call conformation **B**) respect to the large majority of the adsorbed **SubPc-MP** (conformation **A,** Figure 6a). A linescan comparison of conformations **A** and **B** can be found in SI Figure S26. This conformation, **B** (Figure 6b), clearly resembles the single reverse-adsorbed conformation of **SubPc-Azo** and **SubPc-MN**. DFT calculations confirm that, besides a vertical adsorption (conformation **A**, Figure 6c), a reverse adsorption, with the axial ligand flat on the surface and the SubPc base nearly vertical, is energetically possible for **SubPc-MP** (conformation **B**, Figure 6d).

**Figure 6:**
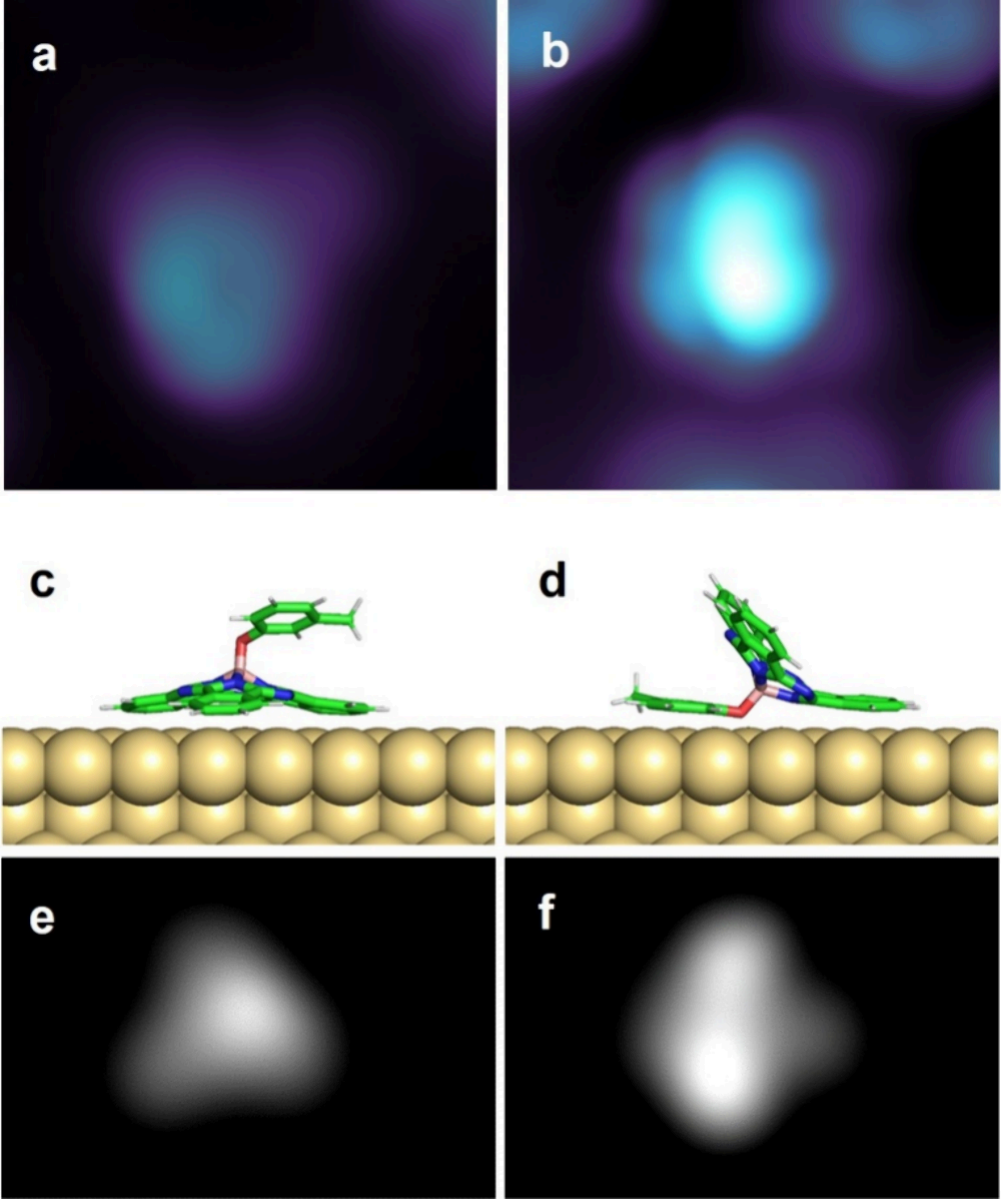
STM images and simulations for the two configurations of **SubPc-MP**. (a) STM image of conformation **A** (*U* = 0.2 V, *I* = 1 pA, size 2.8 nm × 2.8 nm); (b) STM image of conformation **B** (*U* = 0.8 V, *I* = 10 pA, size 2.8 nm × 2.8 nm); (c) adsorption geometry of conformation **A**; and (d) adsorption geometry of conformation **B**. (e) Simulated STM image corresponding to the adsorption geometry in (c), image size 3.0 nm × 2.0 nm; and (f) simulated STM image corresponding to the adsorption geometry in (d), image size 3.0 nm × 2.0 nm.

The corresponding simulated STM images in Figure 6e,f are in very good agreement with the experimental images of Figure 6a,b, confirming the assignment. No chain formation is observed for **SubPc-MP**, probably also because of the very low coverage of conformation **B** on Au(111). The low occurrence of conformation **B** confirms that reducing the π-conjugation length of the axial ligand decreases its interaction with the surface, making the molecule more likely to adopt the standing-up geometry.

The vertically adsorbed **SubPc-MP** in conformation **A** can also be manipulated by the STM tip. By a very soft lateral manipulation, the axial ligand unit of **SubPc-MP** can rotate independently of the SubPc base (Figure 7), showing that rotation around the boron–oxygen bond of the ligand is possible and that the decoupling from the surface has been successfully achieved.

**Figure 7:**
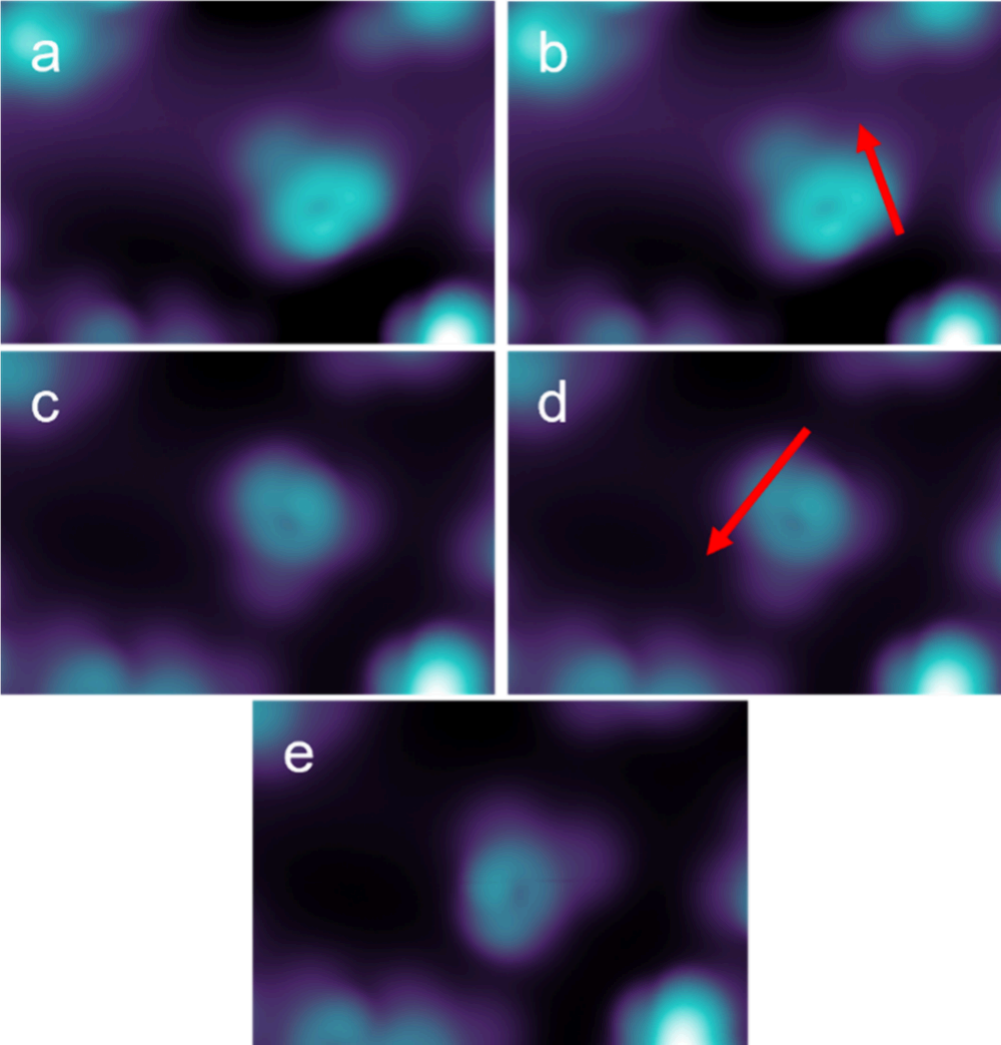
STM image of lateral manipulation sequence of **SubPC-MP** only rotating the methylphenoxy ligand (a–e) (image parameters: *U* = 0.2 V, *I* = 1 pA, 4.9 nm × 3.4 nm) by moving the tip along a fixed trajectory marked with red arrows with constant current and voltage kept constant (lateral manipulation parameter: *U* = 10 mV, *I* = 2 pA).

## Conclusions

In summary, we have designed and investigated SubPc molecules functionalized with axial ligands of different conjugation lengths on the Au(111) surface. We observe that increasing the length of the ligand from 3-methylphenoxy (**SubPc-MP**) to 6-methylnaphthoxy (**SubPc-MN**) and to phenylazophenoxy (**SubPc-Azo**), causes a reverse adsorption of the molecules, where the SubPc base is vertically adsorbed and the conjugated axial ligand strongly interacts with the metal surface. Due to this particular reverse adsorption geometry, **SubPc-Azo** and **SubPc-MN** form 1D lines with different orientations and packing, where the molecules interact with each other by van der Waals (vdW) interaction. The shortest **SubPc-MP,** which has a reduced conjugation in the axial ligand, on the other hand, adsorbs mainly vertically, showing reverse adsorption with very low probability. This indicates that we have reached with **SubPc-MP** the critical length of the axial ligand, allowing a vertical adsorption and rotation.

Our results set the basis for the development of a modular and flexible platform for single-molecule machines on surface based on SubPc and functionalized with rationally designed axial ligands, like, for example, chiral asymmetric structures or switchable units. Furthermore, the flexible design of the axial ligands offers a rational strategy to control the orientation and packing of SubPc-based molecular structures.

## Methods

### Experimental Methods

Experiments were performed using a custom-built LT-STM operating at 5 K and ultrahigh vacuum (*p* ≈ 1 × 10^–10^ mbar). The Au(111)-sample was prepared by sputtering with Ar and annealing at 450 °C for multiple cycles until it was atomically clean. All molecules were deposited with the surfaces kept at room temperature by thermal evaporation by using a commercially available Knudsen-cell evaporator (220–250 °C). Additionally the middle **SubPc-MN** was deposited for comparison on a Au(111) surface kept at 5 K, by flash evaporation from a silicon wafer through rapid heating (Figure S22). All shown images were recorded using constant current mode with the bias voltage applied to the sample.

### Computational Methodology

SubPc bearing different substituents were initially constructed using the BIOVIA Materials Studio software package.^22^ The initial geometries of the molecules were built by employing the standard molecular construction tools provided in the software. Special attention was paid to the arrangement of the substituents in order to minimize steric hindrance prior to further optimization.

The geometrical structures were subsequently optimized within the Forcite module of the Materials Studio. The optimization procedure was performed using the SMART algorithm, which allows for the efficient convergence of molecular geometries. The highest level of optimization quality available in the program, designated as UltraFine, was employed in order to achieve accurate equilibrium structures. The convergence thresholds were chosen to ensure reliable results and were set as follows: an energy convergence tolerance of 2 × 10^–5^ kcal·mol^–1^, a force tolerance of 0.001 kcal·mol^–1^·Å^–1^, and a maximum displacement tolerance of 10^–5^ Å. In order to guarantee full convergence of the system, the maximum number of optimization iterations was fixed at 5000. No external pressure was applied, and optimization of the simulation cell parameters was not performed at this stage, as only the molecular geometry was of interest. The geometry optimization was carried out by employing the universal force field (UFF),^23^ which is widely recognized as a transferable and reliable force field for organic and organometallic systems. Within this framework, atomic charges were assigned consistently according to UFF parametrization.

In the next stage, a simulation cell containing the Au(111) surface was constructed. The gold slab was generated using Build Surface in the Materials Studio. The lateral dimensions of the simulation box were chosen to ensure the presence of a single adsorbed molecule, and the parameters along the a and b directions were set to 58 × 58 Å. A vacuum layer of 100 Å was introduced along the *c* axis, thus preventing spurious interactions between periodic images and creating an effectively infinite slit confined between two immobile parallel Au(111) surfaces. The resulting unit cell was defined as primitive, with the lattice angles fixed at α = β = 90° and γ = 120°. Within the Bravais lattice classification, this corresponds to a triclinic unit cell, which was considered appropriate for the present system. The interactions between the SubPc molecules and gold surface were dispersive according to the Lennard-Jones potential. Interactions among several SubPc molecules include the same Lennard-Jones potential and electrostatic potential.

MD simulations were then performed using the Universal Force Field in the canonical (NVT) ensemble in which the number of atoms, the system volume, and the temperature were held constant throughout the calculations. The simulation temperature was fixed at 228 K, a value corresponding to the experimental deposition temperature of the molecules on the gold surfaces. During the initial stage of the MD procedure, equilibration of the system was performed for approximately 5 ns in order to allow the molecular arrangement and the gold surface to relax toward a steady state. This equilibration stage was followed by a production run of 5 ns, during which trajectory snapshots were collected at regular time intervals for subsequent structural and dynamical analyses. The Nosé thermostat with a *Q* ratio of 0.01 was employed to regulate the temperature during the simulations and to ensure the stability of the NVT ensemble.

For final geometry optimization of the adsorbed states on the surface and STM image simulations, we used the DFT method as implemented in the CP2K software package (cp2k.org) with the Quickstep module.^22^ The Perdew–Burke–Ernzerhof exchange-correlation functional,^23^ the Goedecker-Teter-Hutter pseudopotentials,^24^ and the valence double-ζ basis sets, in combination with the DFT-D2 method of Grimme^25^ for vdW correction, were applied. We used 3 layers of gold, where the upper layer was allowed to be relaxed (planar supercell 29.8 Å × 19.9 Å, vacuum size 60 Å, maximum force 4.5 × 10^–5^ a.u.). The calculated data were analyzed, and the images were generated by the PyMOL Molecular Graphics System, version 2.4 open-source build, Schrödinger, LLC and homemade scripts.

## Supplementary Material

















## Notes

The following files are available free of charge online: The Supporting Information is available free of charge at https://pubs.acs.org/doi/10.1021/acsnano.5c17283.

Details on synthesis and chemical characterization, supplementary STM images and calculations, manipulation experiments (PDF)
Movie M1 showing the complete manipulation sequence of **SubPc-Azo** shown in Figure 2 (PDF)
Molecular dynamics movie M2 showing the adsorption dynamic of **SubPc-MN** on Au(111) (MP4)
Molecular dynamics movie M3 showing the formation of an ensemble of four **SubPc-MN** on Au(111) (MP4)
Molecular dynamics movie M4 showing a chain of **SubPc-MN** on Au(111) (side view) (MP4)
Molecular dynamics movie M5 showing a chain of **SubPc-MN** on Au(111) (top view) (MP4)
Molecular dynamics movie M6 showing a chain of **SubPc-Azo** on Au(111) (top view) (MP4)
Movie M7 presenting the isolation of a reverse-adsorbed **SubPc-MP** from an ensemble of five molecules (PDF)

## ∥

Felix-Bloch-Institute of Solid-State Physics, Faculty of Physics and Earth Sciences, Universität Leipzig, Linnéstrasse 5, Leipzig 04103, Germany
