# Supplementary material for: Subphthalocyanine Platform for Single-Molecule Machines on Surface: Ligand-Directed Adsorption on Au(111)
Source: ACS Nano. 2026 Mar 12;20(11):9139–46. doi: 10.1021/acsnano.5c17283 (PMC13019658; doi:10.1021/acsnano.5c17283)
Supplement: Supplementary file 1 [file nn5c17283_si_001.pdf]

Supporting Information

A Subphthalocyanine Platform for Single-Molecule Machines on Surface: Ligand Directed Adsorption on Au(111)

Franz Plate[1‡], Soyoung Park[2,3‡], Ebru Cihan[1], Natasha Khera[1], Ningwei Sun[2], Pranjit Das[1], Olga Guskova[2], Dmitry A. Ryndyk[1,2], Franziska Lissel[2,3], Francesca Moresco[1*]*

[1]Center for Advancing Electronics Dresden, TU Dresden, 01062 Dresden, Germany

[2]Leibniz Institute of Polymer Research Dresden, Hohe Strasse 6, 01069 Dresden, Germany

[3]Institute for Applied Polymer Physics, TU Hamburg, 21073 Hamburg, Germany

Table of Contents

(1) Experimental methods and instrumentation ... 3

Nuclear magnetic resonance (NMR) spectroscopy ... 3

Raman spectroscopy ... 3

Electrospray ionization high-resolution mass spectrometry (ESI-HRMS) ... 3

Thermogravimetric analysis (TGA) ... 3

Ultraviolet-visible (UV/Vis) spectroscopy ... 3

(2) Synthesis ... 4

Materials ... 4

4-Phenylazophenoxyboronsubphthalocyanine (SubPc-Azo) ... 4

6-Methylnaphthoxyboronsubphthalocyanine (SubPc-MN) ... 5

3-Methylphenoxyboronsubphthalocyanine (SubPc-MP) ... 5

(3) Experimental results ... 7

NMR, UV-Vis, Raman and TGA ... 7

(4) Additional STM experiments ... 15

Manipulation of a SubPc-Azo chain ... 15

Overview of SubPc-Azo and SubPc-MN with visible surface reconstruction ... 16

Manipulation of a single SubPc-MN ... 17

Isolation of a single SubPc-MN molecule ... 18

Isolation of a single SubPc-Azo molecule ... 19

Lateral Manipulation of SubPc-Azo chain ... 19

Deposition on Au(111) kept at 5K surface SubPc-MN ... 20

Co-deposition of SubPc-MN and pure SubPc ... 21

Overview of SubPc-MP on Au(111) ... 22

Isolation of reverse adsorbed SubPc-MP ... 23

Comparison of different single SubPc-MP conformations ... 24

Rotation of SubPc-MP rotor unit through lateral manipulation ... 25

Linescan comparison SubPc-Azo, SubPc-MN and SubPc-MP ... 26

Linescan comparison SubPc-Azo, SubPc-MN and SubPc-MP ... 27

(5) Additional DFT calculations and simulated images ... 28

SubPc-MN DFT calculations and simulated images ... 28

Comparison theoretical images of SubPC-chains to experimental images ... 29

(6) References ... 30

Experimental methods and instrumentation

Nuclear magnetic resonance (NMR) spectroscopy

NMR spectra were obtained with a Bruker Avance III 500 spectrometer, functioning at frequencies of 500.13 MHz for ^{1}H and 125.77 MHz for ^{13}C, respectively. All measurements were carried out at room temperature using deuterated chloroform ($CDCl_3$) as the solvent. Solvent signals were referenced according to the published literature. ($CDCl_3$; ^{1}H $\delta = 7.26$ ppm, ^{13}C $\delta = 77.16$ ppm). Signal multiplicities were represented by conventional abbreviations: s for singlet, d for doublet, q for quartet, and m for multiplet.

Raman spectroscopy

Raman measurements were carried out on a WITec alpha300 R confocal Raman microscope using a 20x objective. Three excitation wavelengths (532 nm, 633 nm and 785 nm) were employed at respective laser powers of 1 µW, 0.2 mW and 0.5 mW. Spectra were recorded with an integration time of 0.5 s per acquisition and 500 co-added accumulations to enhance the signal-to-noise ratio. Post-acquisition processing was limited to a linear baseline correction.

Electrospray ionization high-resolution mass spectrometry (ESI-HRMS)

The ESI-HRMS spectra were collected utilizing a Waters XEVO G2X QTOF in positive ionization mode. A mass range of m/z 50 to 1200 was chosen to determine the precise mass of the sample's molecular ion and its fragmented ions.

Thermogravimetric analysis (TGA)

TGA Q 5000 from TA Instruments was used for thermogravimetric analysis, and FTIR spectra were recorded on NICOLET IS20 MID-IR DTGS Spektrometer from Thermo Fisher. The thermal degradation investigations were conducted under a nitrogen flow. The furnace was constantly heated up to 800 °C at a heating rate of 10 °C/min.

Ultraviolet-visible (UV/Vis) spectroscopy

UV-Vis absorption spectra were measured using a Cary 5000 UV/Vis/NIR spectrophotometer (Agilent Technologies Deutschland GmbH) equipped with a 1 cm path-length quartz cuvette. Spectroscopic measurements were performed in the wavelength range from 800 nm to 250 nm.

Synthesis

Materials

Unless otherwise indicated, all compounds were purchased from commercial vendors. All chemicals were used without further purification unless it is stated. Molecular weights were calculated taking into account the purities specified by the respective suppliers.

4-Phenylazophenoxyboronsubphthalocyanine (SubPc-Azo)

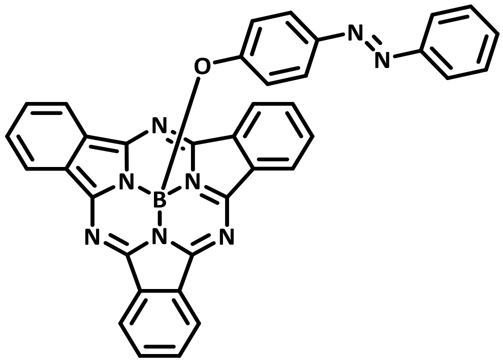

This compound was synthesized using a modified procedure from the literatures[1,2].

Boron subphthalocyanine chloride (0.7 g, 1.38 mmol) was added to a 2-neck round-bottom flask equipped with a reflux condenser. The flask was evacuated and backfilled with argon three times (pump–fill cycle) to ensure an inert atmosphere. Dry toluene (50 mL) and 4-phenylazophenol (1.4 g, 6.92 mmol) were then added under a constant argon flow. The reaction mixture was stirred and heated to reflux for 20 hours. The reaction's completion was observed using thin-layer chromatography (TLC). After completion, the crude mixture was filtered through a celite pad to remove inorganic dye impurities originating from the commercial boron subphthalocyanine chloride. Subsequently, the filtrate was concentrated under reduced pressure. The crude product was purified by dry column vacuum chromatography (DCVC), using a gradient elution from 5% to 50% dichloromethane in *n*-hexane, maintaining the final concentration. The product was collected and recrystallized in a DCM/n-hexane mixture to afford the target molecule as dark magenta solid. (0.79 g, 96%)

^{1}H NMR (500 MHz, $CDCl_3$) δ = 8.88 (m, 6H), 7.93 (m, 6H), 7.74 (d, 2H), 7.44–7.37 (m, 5H), 5.50 (2H); **^{13}C NMR** (125 MHz, $CDCl_3$) δ = 155.82, 152.84, 151.58, 147.40, 131.18, 130.41, 130.10, 129.07, 124.20, 122.61, 122.44, 119.45; **Raman** (633 nm) $\tilde{\nu}$ (cm^{-1}) = 502, 635, 700, 745, 779, 913, 1131, 1197, 1287, 1325, 1348, 1402, 1431, 1456, 1486; **ESI-HRMS** m/z calcd for [M + H^+] 593.2004 found 593.2015; **UV-Vis** (nm) 310, 563.

6-Methylnaphthoxyboronsubphthalocyanine (SubPc-MN)

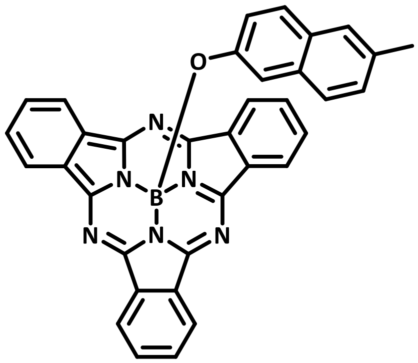

This compound was synthesized using a modified procedure from the literature[1,2].
Boron subphthalocyanine chloride (0.15 g, 0.30 mmol) was added to a 2-neck round-bottom flask equipped with a reflux condenser. The flask was evacuated and backfilled with argon three times (pump–fill cycle) to ensure an inert atmosphere. Dry toluene (10 mL) and 6-methyl-2-naphthol (0.2 g, 1.20 mmol) were then added under a constant argon flow. The reaction mixture was stirred and heated to reflux overnight. After completion, the crude mixture was filtered through a celite pad to remove inorganic dye impurities originating from the commercial boron subphthalocyanine chloride. Subsequently, the filtrate was concentrated under reduced pressure. The crude product was purified by dry column vacuum chromatography (DCVC), using a gradient elution from 0.5% to 10% dichloromethane in n-hexane, maintaining the final concentration. The product was collected and recrystallized in a DCM/*n*-hexane mixture to afford the target molecule as dark magenta solid. (0.099 g, 61%)

^{1}H NMR (500 MHz, $CDCl_3$) δ = 8.86 (m, 6H), 7.91 (m, 6H), 7.27-7.25 (m, 2H), 7.14 (d, 1H), 7.09 (d, 1H) 5. 68 (s, 1H), 5.63 (d, 2H), 2.35 (s, 3H); **^{13}C NMR** (125 MHz, $CDCl_3$) δ = 151.55, 149.83, 133.26, 132.30, 131.18, 129.95, 129.49, 128.19, 128.07, 126.64, 126.41, 122.38, 120.90, 114.17, 21.51; **Raman** (785 nm) $\tilde{\nu}$ (cm^{-1}) = 508, 645, 702, 746, 782, 1139, 1198, 1291, 1327, 1348, 1408, 1457; **ESI-HRMS** m/z calcd for [M + H^+] 553.1943 found, 553.1955; **UV-Vis** (nm) 305, 563.

3-Methylphenoxyboronsubphthalocyanine (SubPc-MP)

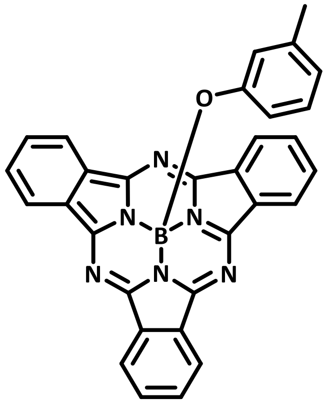

This compound was synthesized using a modified procedure from the literature [1,2].
Boron subphthalocyanine chloride (0.2 g, 0.39 mmol) was added to a 2-neck round-bottom flask equipped with a reflux condenser. The flask was evacuated and backfilled with argon three times (pump–fill cycle) to ensure an inert atmosphere. Dry toluene (10 mL) and *m*-cresol (0.3 mL, 2.74 mmol) were then added under a constant argon flow. The reaction mixture was stirred and heated to reflux overnight. After completion, the crude mixture was filtered through a celite pad to remove inorganic dye impurities originating from the commercial boron subphthalocyanine chloride. Subsequently, the filtrate was concentrated under reduced pressure. The crude product was purified by dry column vacuum chromatography (DCVC), using a gradient elution from 0.5% to 10% dichloromethane in n-hexane, maintaining the final concentration. The product was collected and recrystallized in a DCM/*n*-hexane mixture to afford the target molecule as dark magenta solid. (0.150 g, 76%)

^{1}H NMR (500 MHz, $CDCl_3$) δ = 8.86 (m, 6H), 7.91 (m, 6H), 6.63 (t, 1H), 6.43 (d, 1H), 5.25 (s, 1H), 5.15 (d, 1H), 1.94 (s, 3H); **^{13}C NMR** (125 MHz, $CDCl_3$) δ = 152.63, 151.53, 138.89, 131.17, 129.93, 128.66, 122.38, 122.35, 120.09, 115.74, 21.16; **Raman** (785 nm) $\tilde{\nu}$ (cm^{-1}) = 348, 508, 639, 702, 748, 781, 1022, 1133, 1197, 1288, 1324, 1402, 1453; **ESI-HRMS** m/z calcd for [M + H^+] 503.1786 found, 503.1795; **UV-Vis** (nm) 306, 563.

Experimental results

NMR, UV-Vis, Raman and TGA

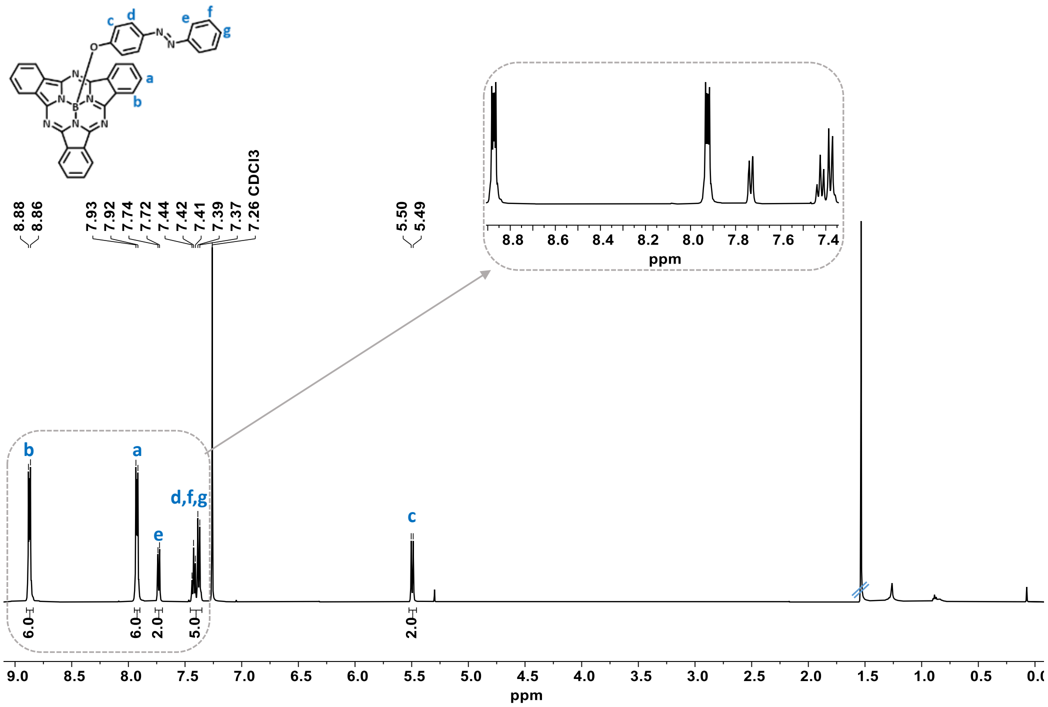

Figure S1. ^{1}H NMR spectrum of (SubPc-Azo) in $CDCl_3$.

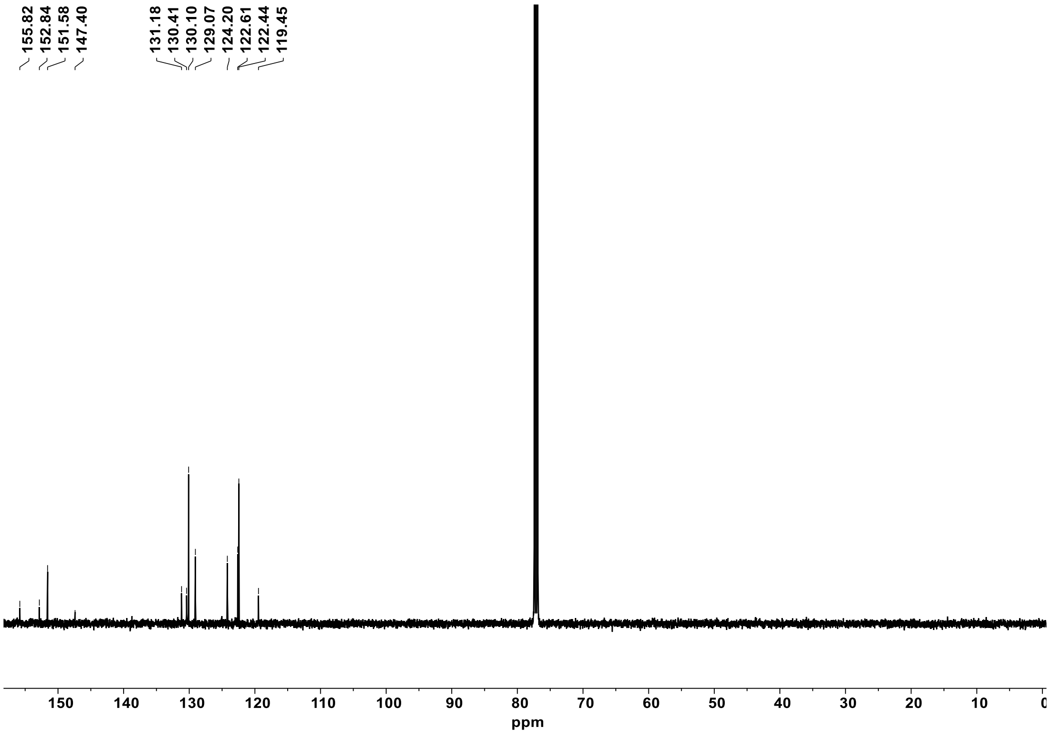

Figure S2. ^{13}C NMR spectrum of (SubPc-Azo) in $CDCl_3$.

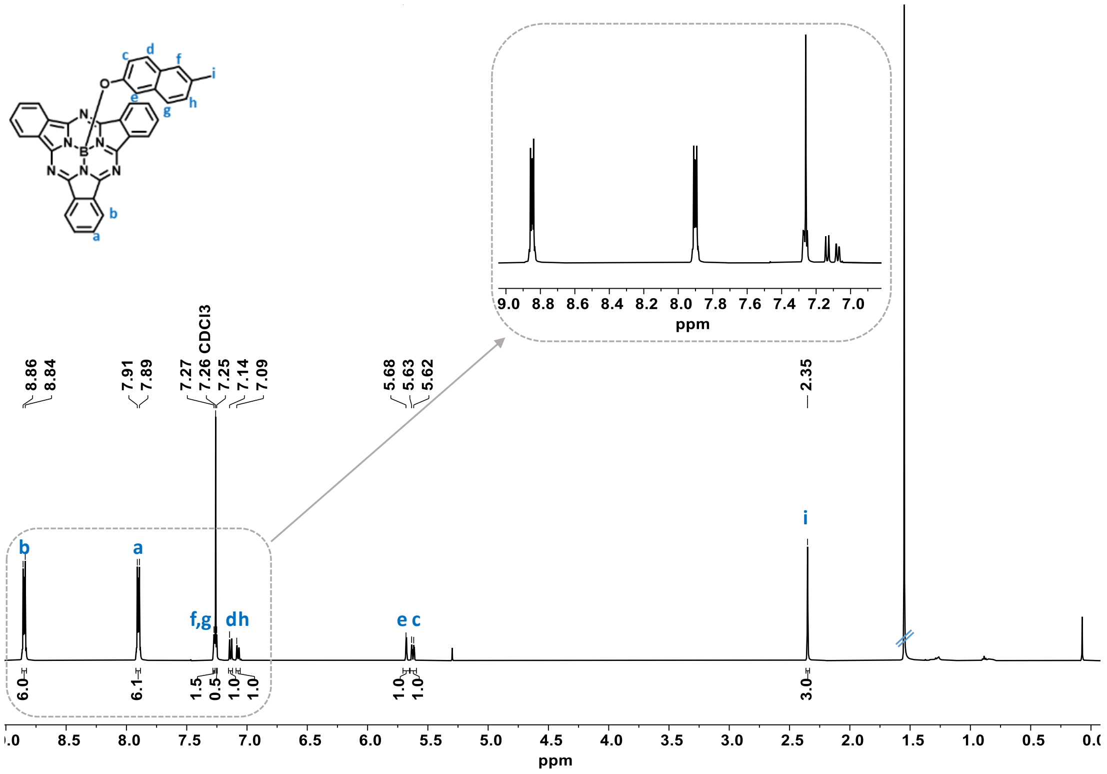

Figure S3. ^{1}H NMR spectrum of (SubPc-MN) in $CDCl_3$.

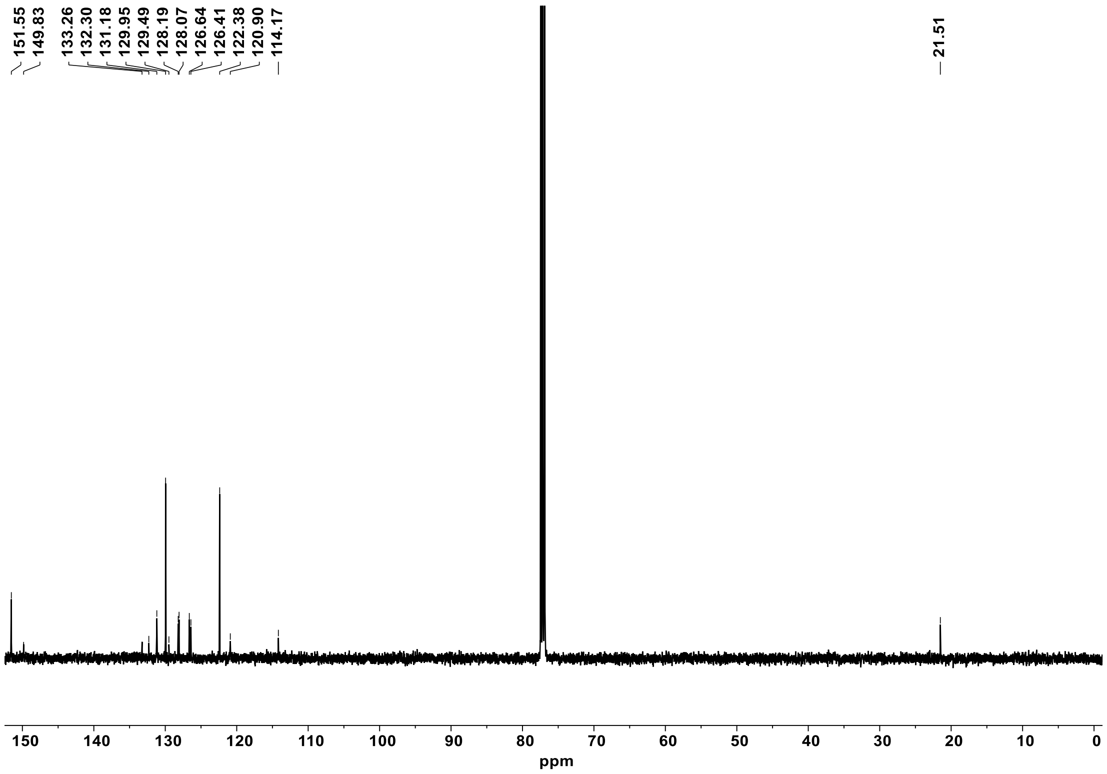

Figure S4. ^{13}C NMR spectrum of (SubPc-MN) in $CDCl_3$.

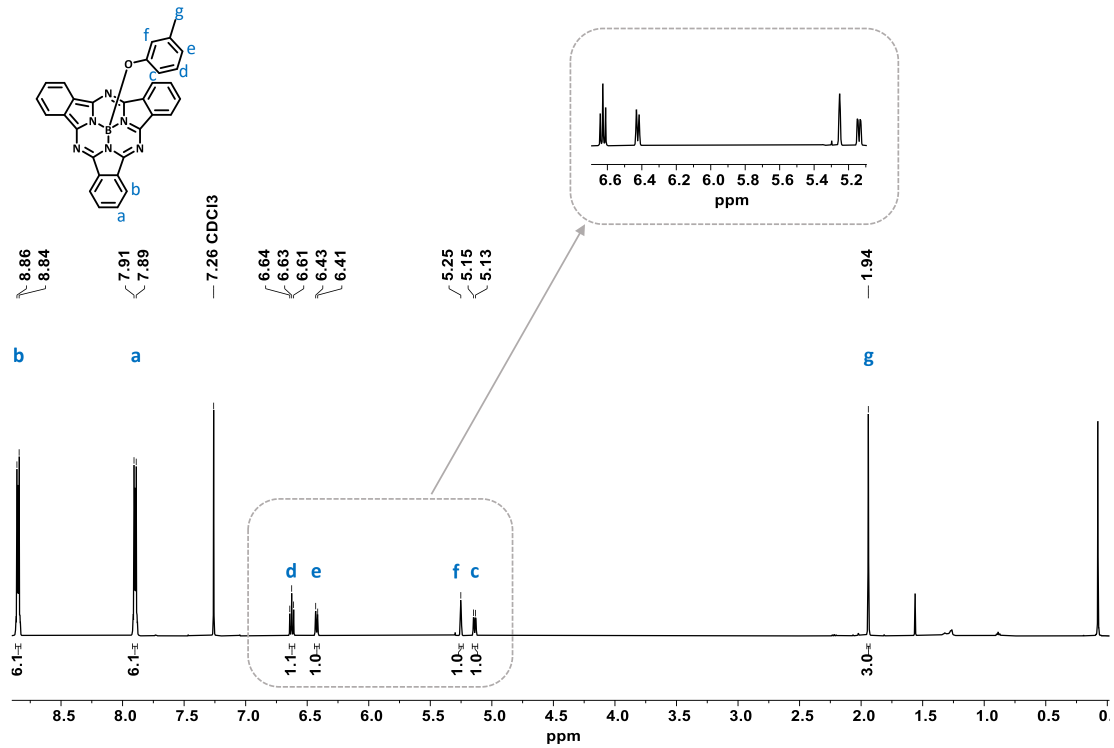

Figure S5. ^{1}H NMR spectrum of (SubPc-MP) in $CDCl_3$.

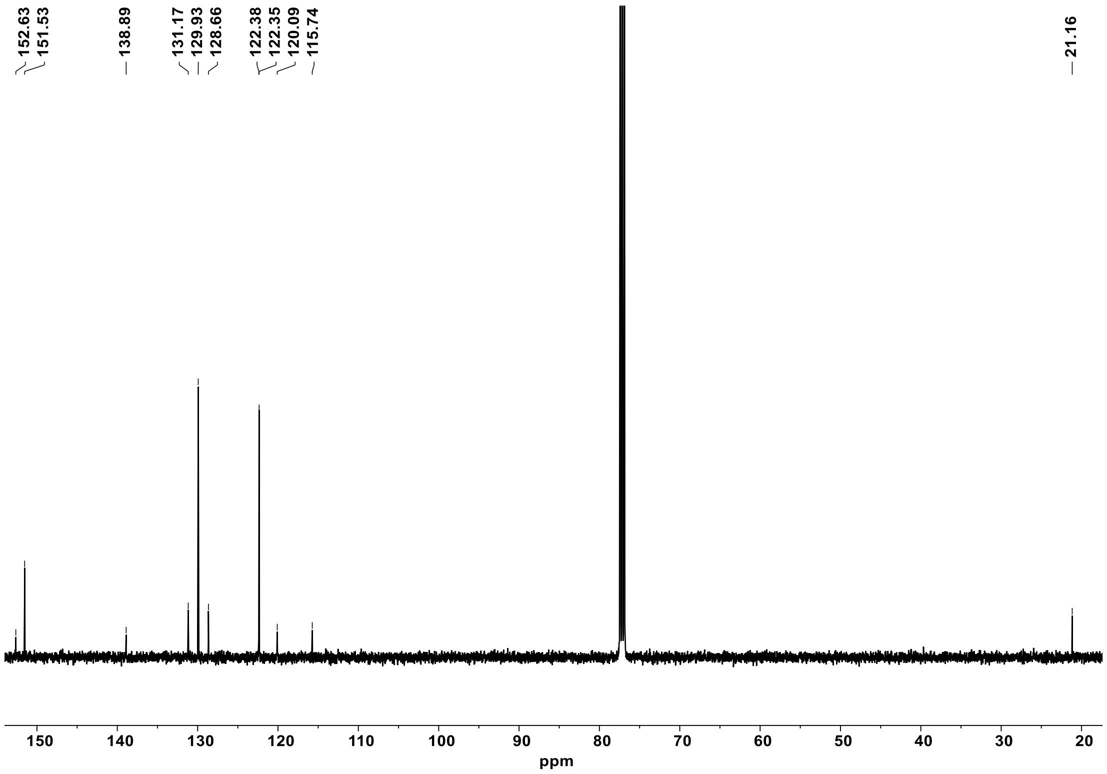

Figure S6. ^{13}C NMR spectrum of (SubPc-MP) in $CDCl_3$.

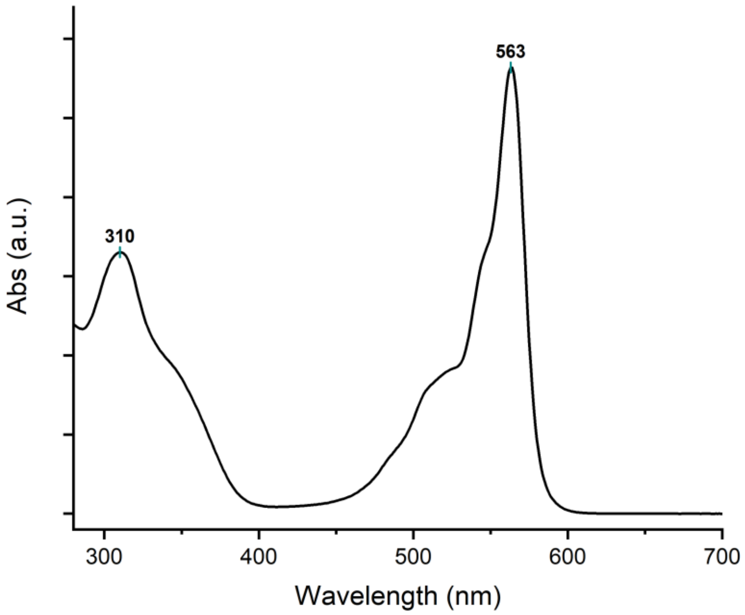

Figure S7. UV-Vis spectrum of (SubPc-Azo).

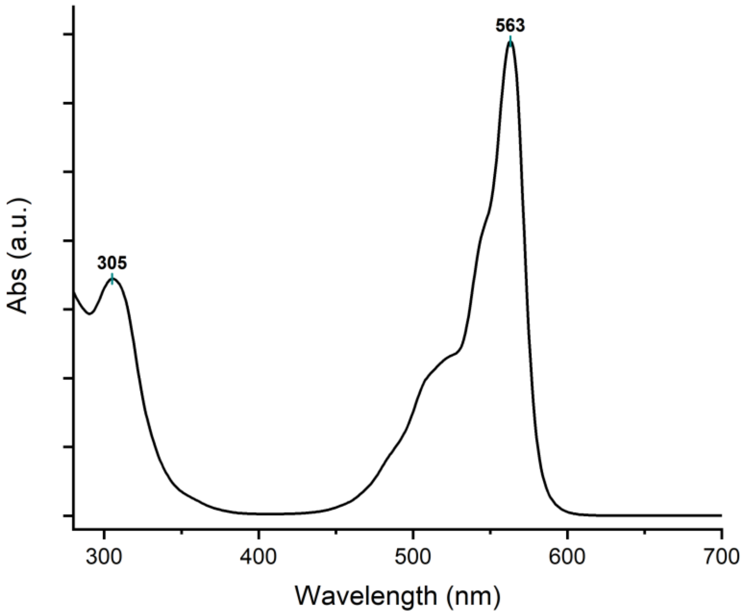

Figure S8. UV-Vis spectrum of (SubPc-MN).

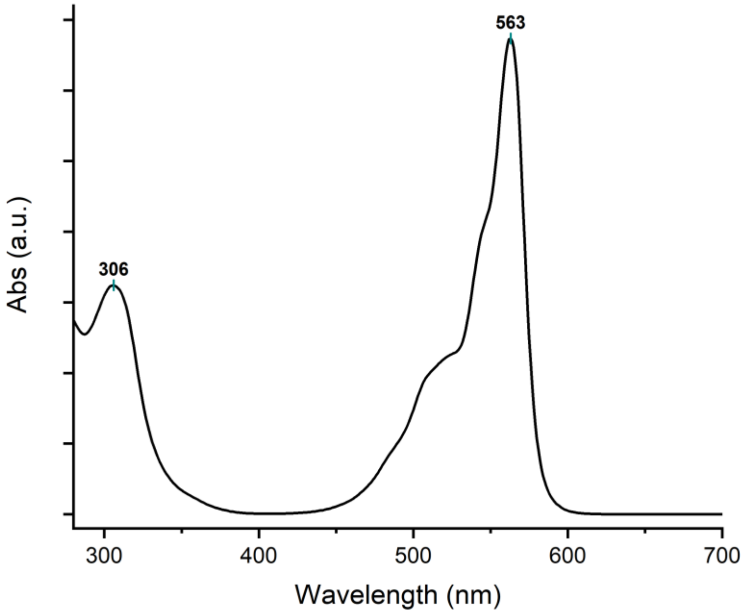

Figure S9. UV-Vis spectrum of (SubPc-MP).

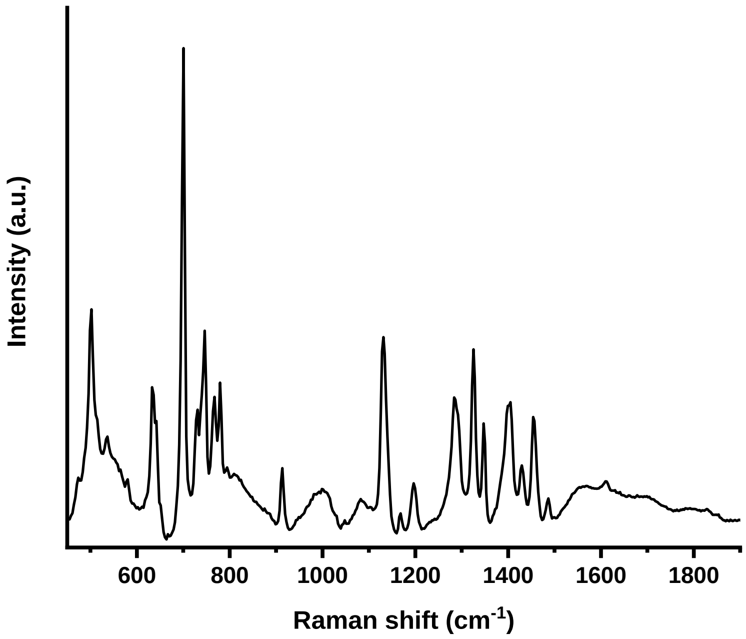

Figure S10. Raman spectrum of (SubPc-Azo).

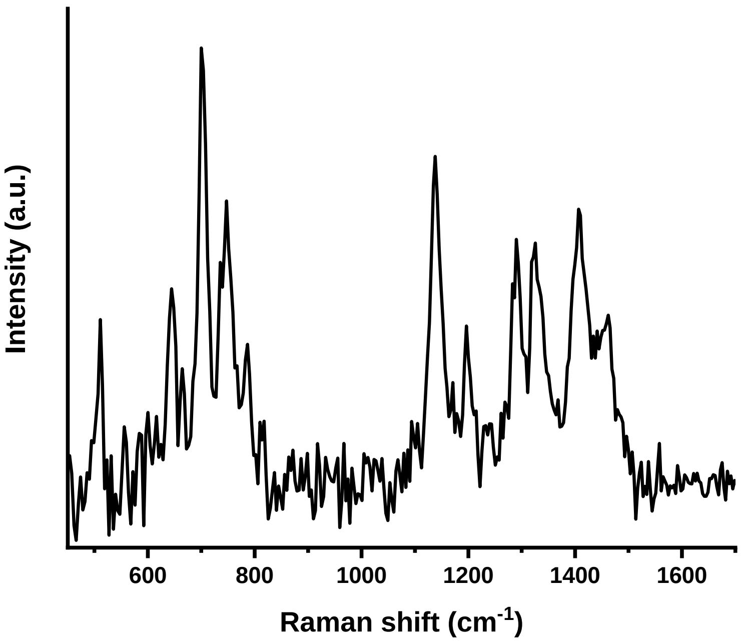

Figure S11. Raman spectrum of (SubPc-MN).

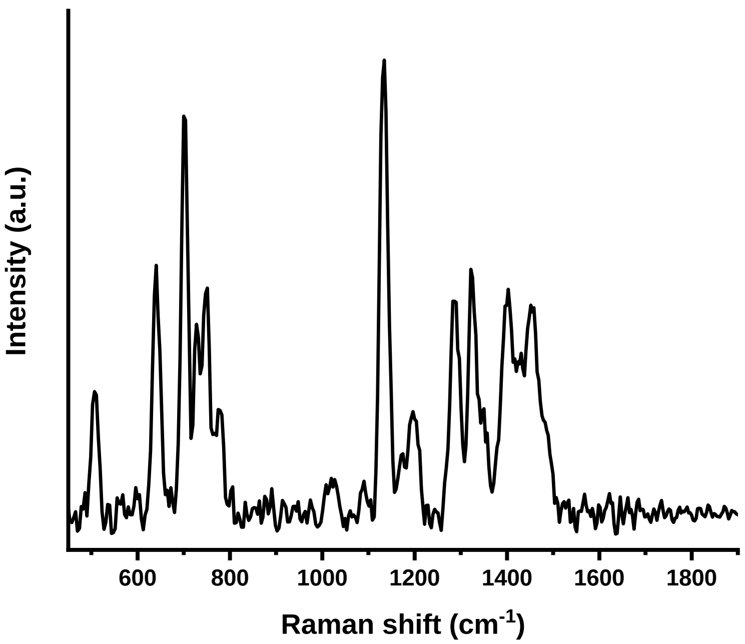

Figure S12. Raman spectrum of (SubPc-MP).

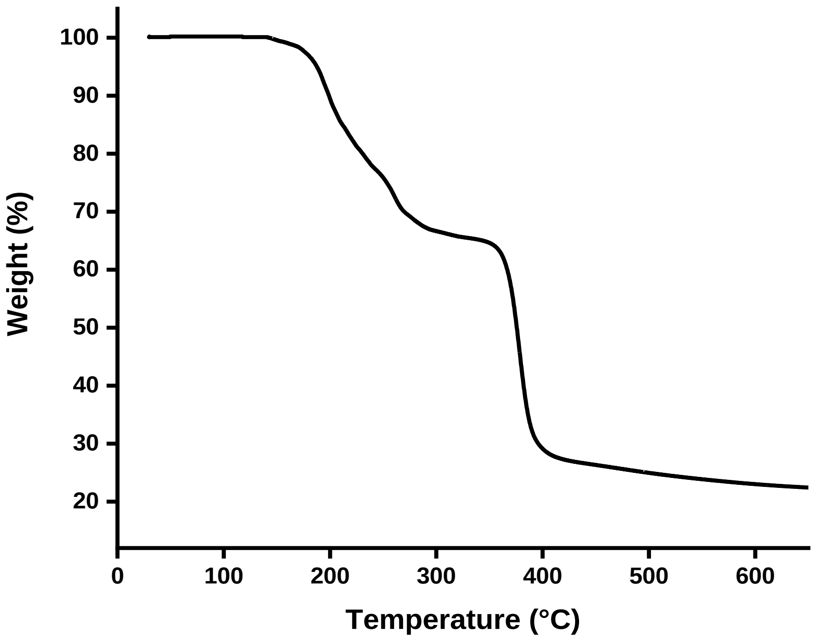

Figure S13. TGA spectrum of (SubPc-Azo).

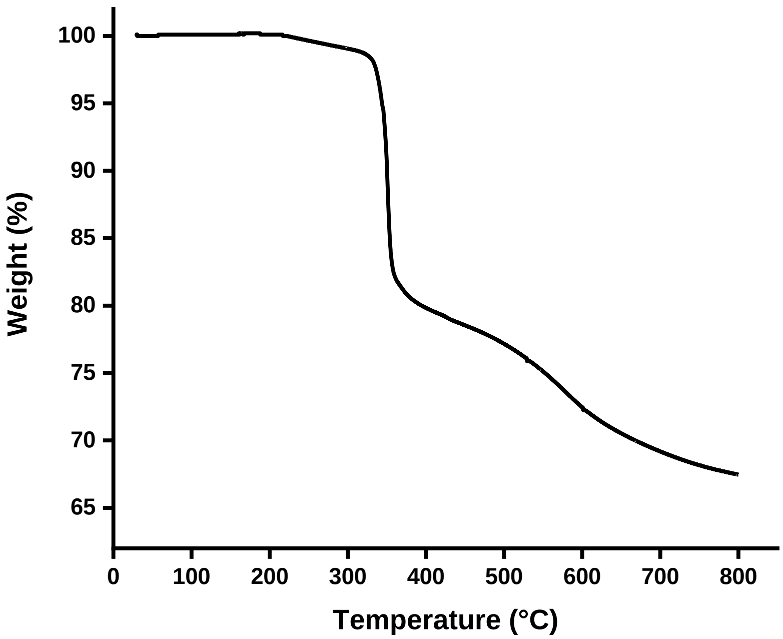

Figure S14. TGA spectrum of (SubPc-MN).

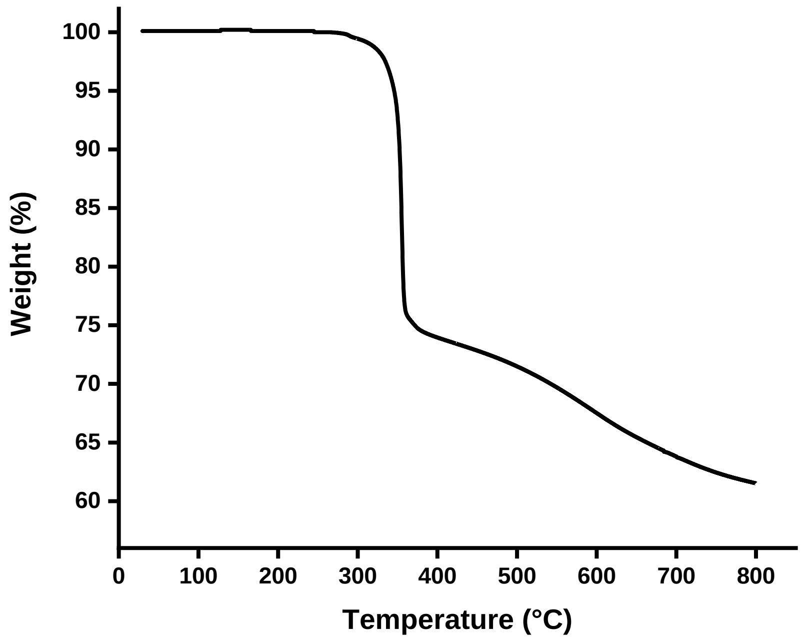

Figure S15. TGA spectrum of (SubPc-MP).

Additional STM experiments
Manipulation of a SubPc-Azo chain

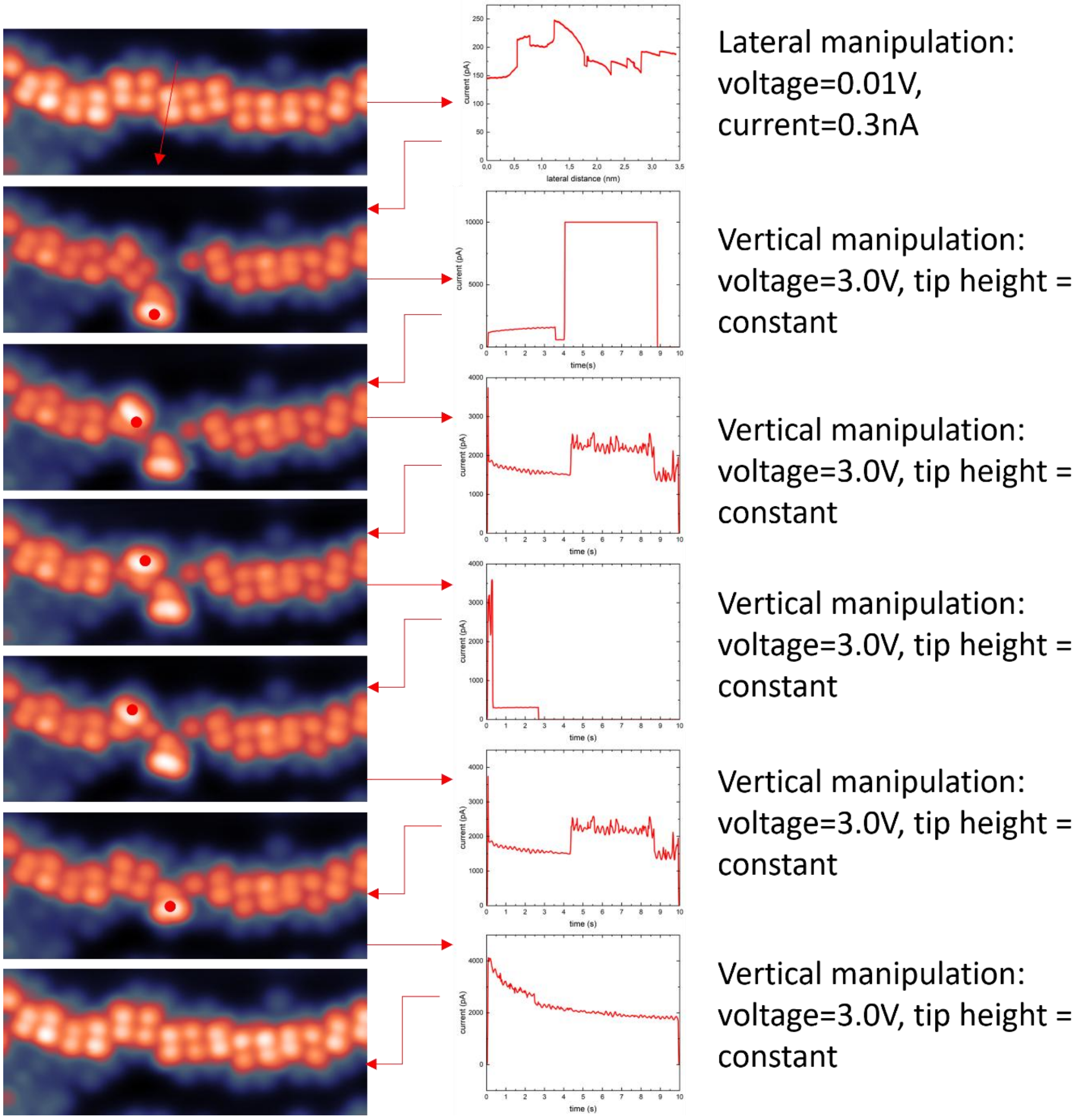

Figure S16. Complete manipulation sequence of a SubPc-Azo chain (Image Parameters: U = 0.5 V, 10 pA, size 10 nm x 4 nm).

Overview of SubPc-Azo and SubPc-MN with visible surface reconstruction

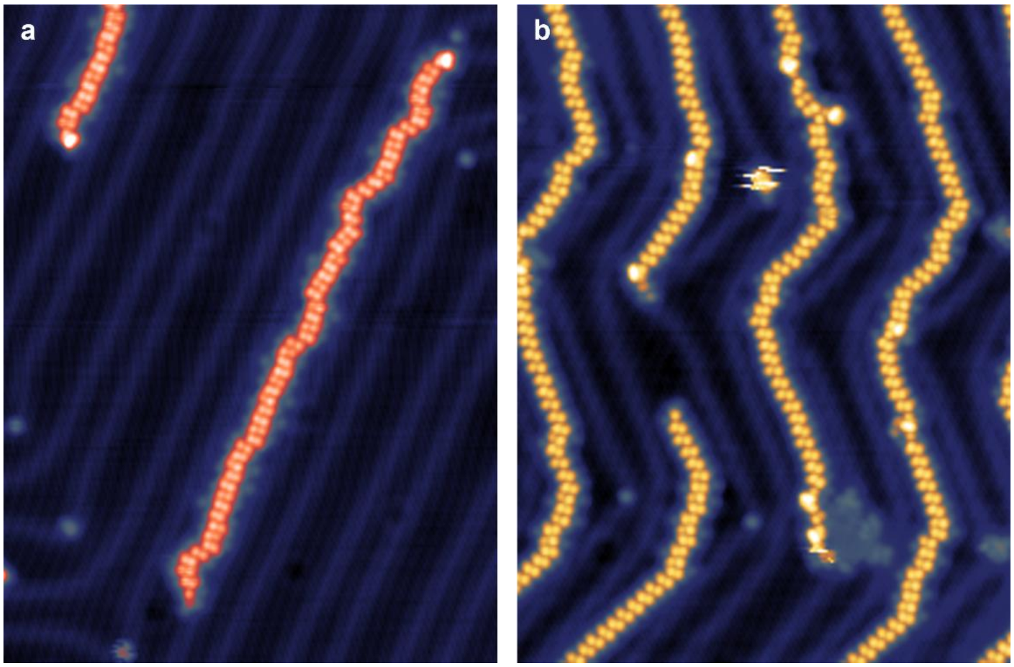

Figure S17. Overview STM images of (a) SubPc-Azo chains (Image parameters: U = 0.5 V, I = 10 pA, size = 30 nm x 40 nm) and (b) SubPc-MN (Image parameters: U = 0.2 V, I = 20 pA, size 30 nm x 40 nm) chains with visible Au(111) herringbone reconstruction.

Manipulation of a single SubPc-MN

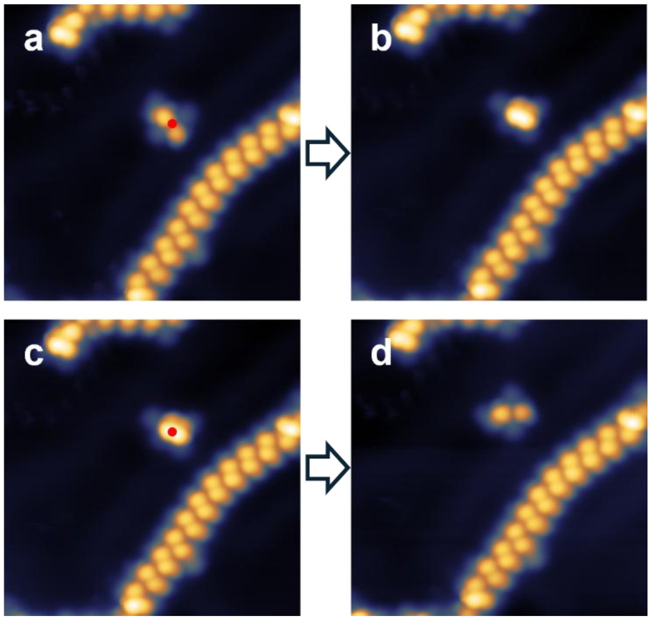

Figure S18. Example of manipulation of single SubPc-MN molecule. (a) Pulsing a single molecule with two phenyl rings pointing up; (b) post pulse single molecule is now only facing one phenyl ring up; (c) pulsing one single molecule with only one phenyl ring facing up switching it briefly back to (d) two phenyl rings pointing up (Image parameters: U = 0.2 V, I = 10 pA, size 10 nm x 10 nm) (pulsing parameters: U = 2.0 V, constant height).

Isolation of a single SubPc-MN molecule

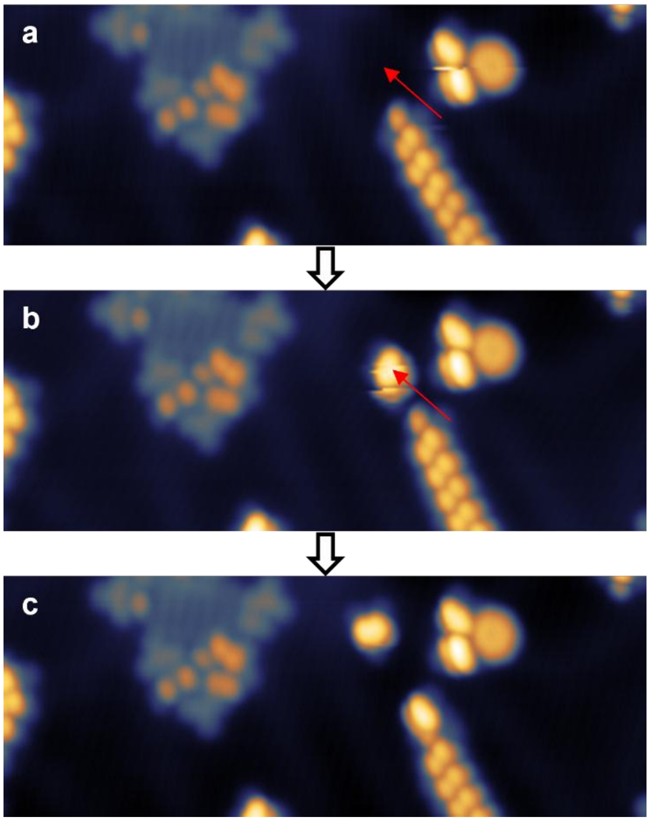

Figure S19. Lateral manipulation of SubPc-MN from the end of a chain. (a) Start isolating from chain through slide tip trajectory from the side; (b) additional manipulation to isolate the molecule completely from neighboring defect and adjacent molecules, which leads to no change in conformation, (c) isolated SubPC-MN molecule; (Image parameters: U = 0.2 V, I = 20 pA, size 20 nm x 7.5 nm) (lateral manipulation parameters: U = 0.01 V, I = 0.15 nA, constant current).

Isolation of a single SubPc-Azo molecule

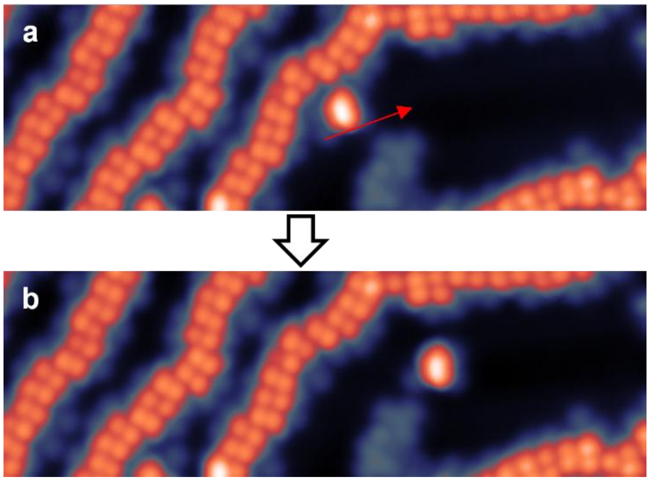

Figure S20. Lateral manipulation of a single SubPc-Azo molecule adjacent to the chain (a) to be without any adjacent defect or molecule (b). (Image parameters: U = 0.5 V, I = 10 pA, size 20 nm x 6.4 nm) (lateral manipulation parameters: U = 0.01 V, I = 0.05 nA, constant current)

Lateral Manipulation of SubPc-Azo chain

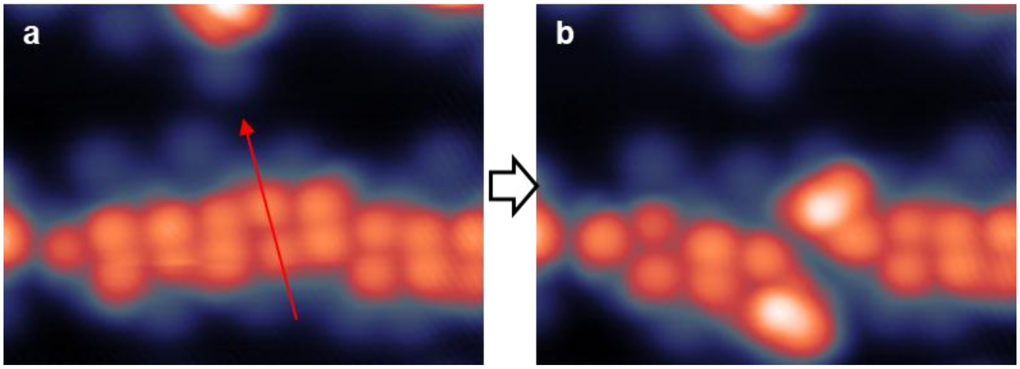

Figure S21. Lateral manipulation across a SubPc-Azo chain (a), which results in a small separation of the molecules building up the chain, leading to a conformation change of the separated molecules (b). (Image parameters: U = 0.5 V, I = 10 pA, size 10 nm x 4.5 nm) (lateral manipulation parameters: U = 0.01 V, I = 0.23 nA, constant current)

Deposition on Au(111) kept at 5K surface SubPc-MN

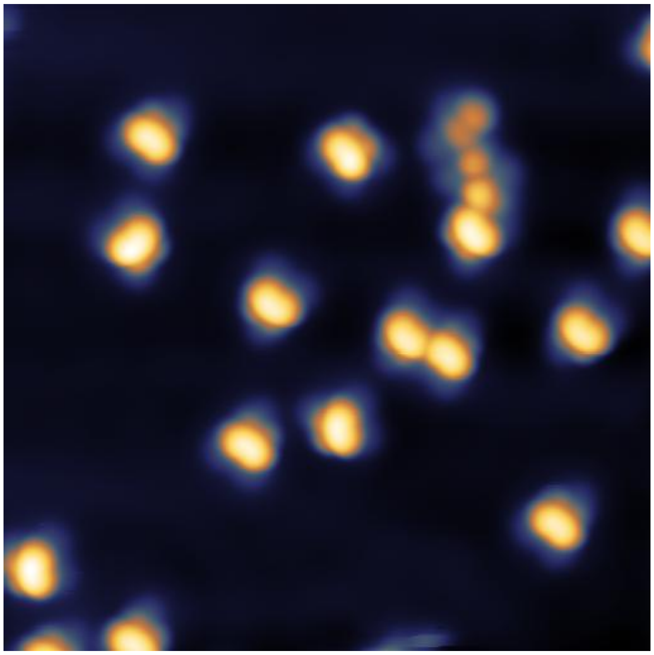

Figure S22. STM image overview of **SubPc-MN** after flash evaporation from Si-Wafer onto the Au(111) surface kept at 5K, a lot of single molecules are visible with a few starting to form the chain conformation. Images parameters: U = 0.2 V, I = 1 pA, size 15 nm x 15 nm

Co-deposition of SubPc-MN and pure SubPc

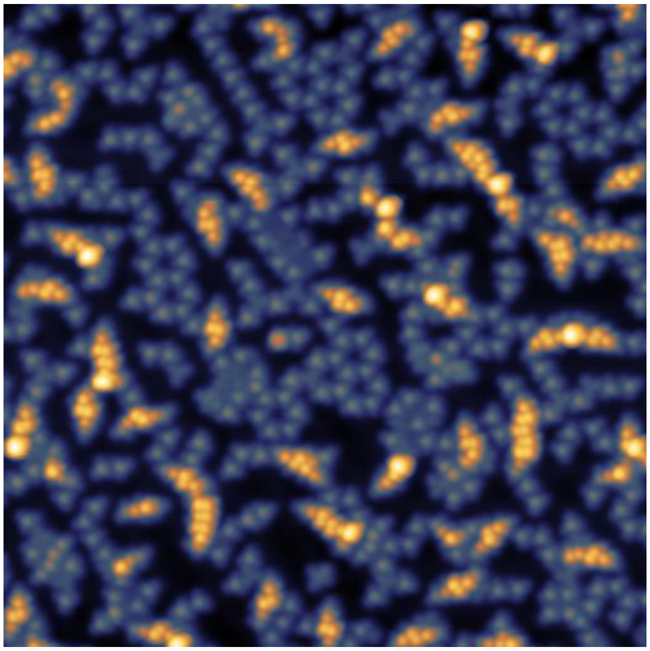

Figure S23. STM image of codeposited pure SubPc and SuPc-MN, both molecules were subsequently deposited by thermal evaporation (220°C-250°C) with the pure Boron subphthalocyanine chloride deposited first, pure SubPc forming ordered hexagonal assembly's and **SubPC-MN** one-dimensional chains (Imaging parameters: U = 0.5 V, I = 30 pA , size 40 nm x 40 nm)

Overview of SubPc-MP on Au(111)

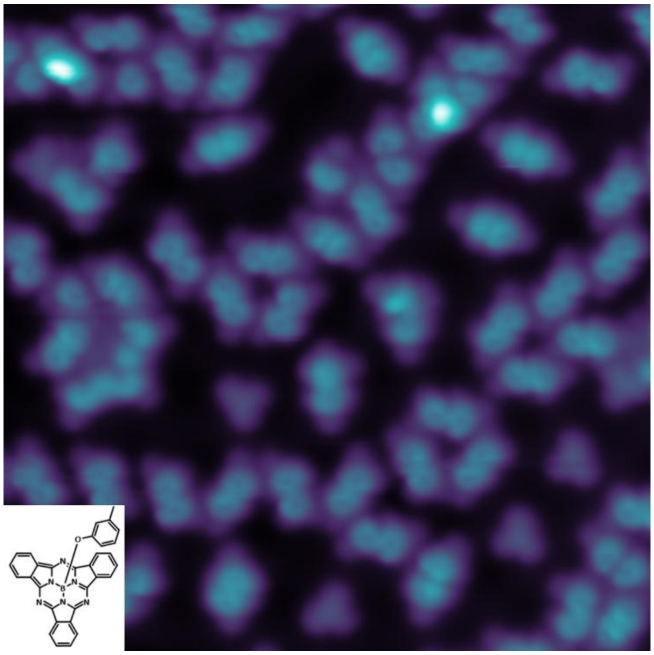

Figure S24. STM image overview after thermal evaporation of **SubPc-MP** from Knudsen cell onto the Au(111) surface (Image Parameters: U = 0.2 V, I = 1 pA, 20 nm x 20 nm)

Isolation of reverse adsorbed SubPc-MP

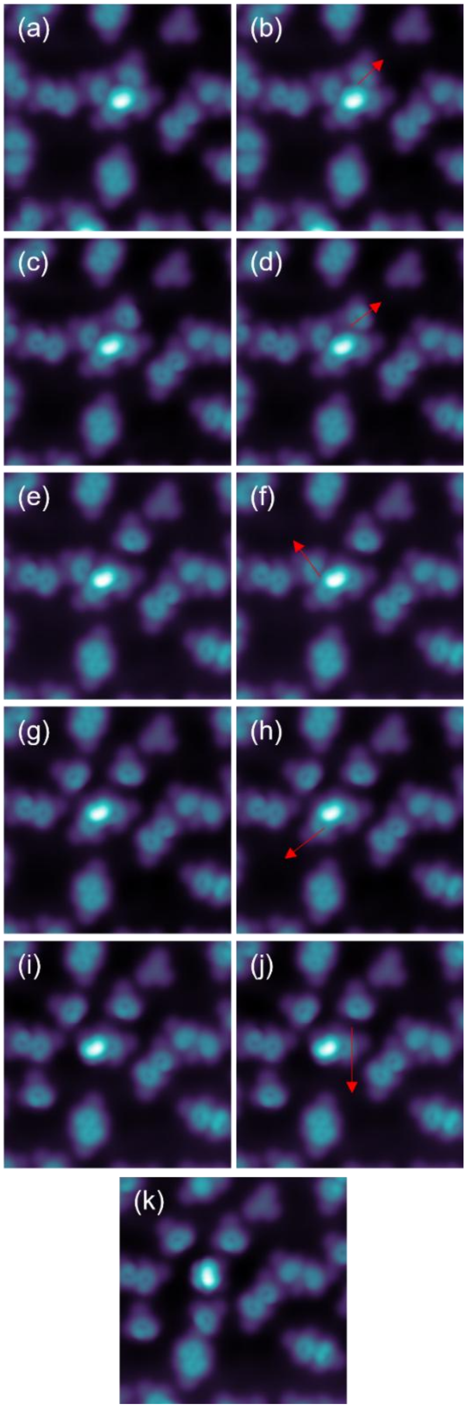

Figure S25. STM images of lateral manipulation sequence to isolate center reverse adsorbed molecule **B** from original pentamer assembly (a-k) (Imaging parameters: U = 0.2 V, I = 1 pA, size 9.85 nm x 9.85 nm, lateral manipulation parameters: 1 pA, 10 mV).

Comparison of different single SubPc-MP conformations

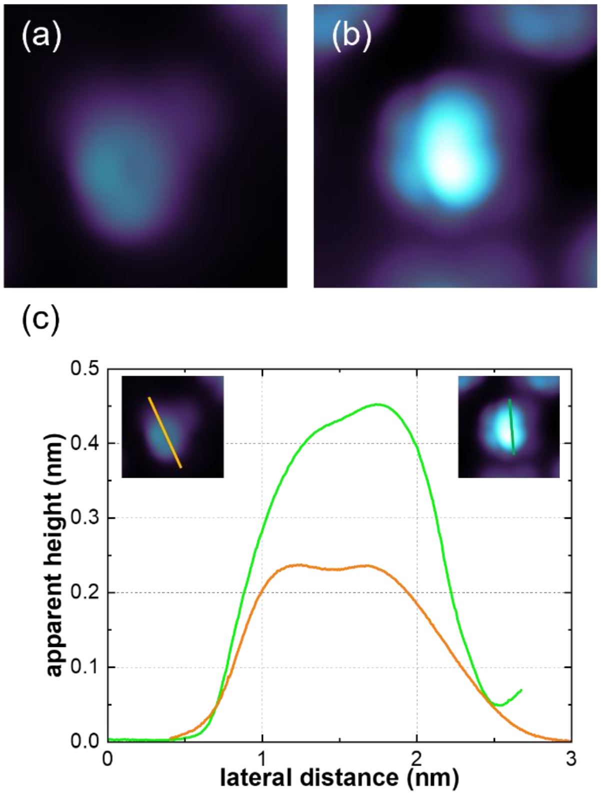

Figure S26. Comparison of SubPc-MP: (a) SubPc-MP in configuration [A] with rotor unit facing up (Image Parameters: U = 0.8 V, I = 10 pA, 2.8 nm x 2.8 nm); (b) SubPc-MP in configuration [B] with rotor unit facing Down (Image Parameters: U = 0.2 V, I= 1 pA, 2.8 nm x 2.8 nm); (c) linescan comparison of (a) and (b)

Rotation of SubPc-MP rotor unit through lateral manipulation

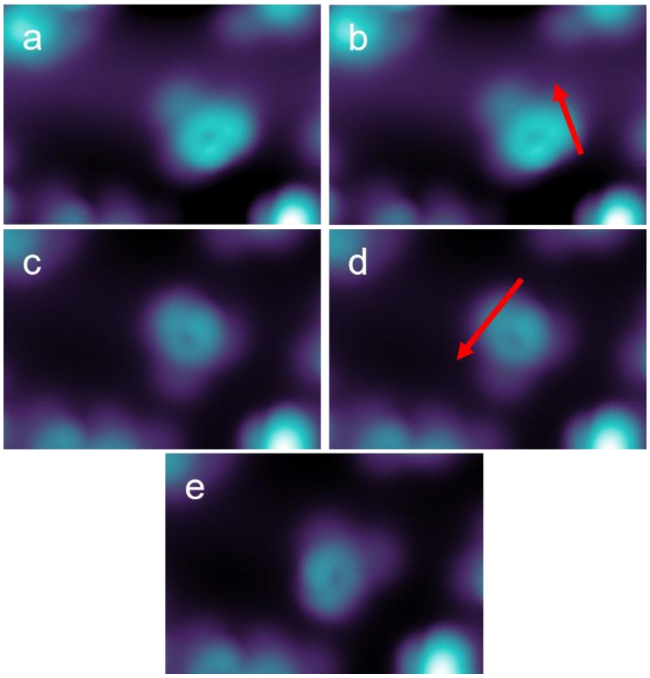

Figure S27. STM image of lateral manipulation sequence of **SubPC-MP** only rotating the methylphenoxy ligand (a-e) (Image Parameters: U = 0.2 V, I= 1 pA, 4.9 nm x 3.4 nm) by moving the tip along a fixed trajectory marked with red arrows with constant current and voltage kept constant. (lateral manipulation parameter: U = 10 mV, I = 2 pA).

Linescan comparison SubPc-Azo, SubPc-MN and SubPc-MP

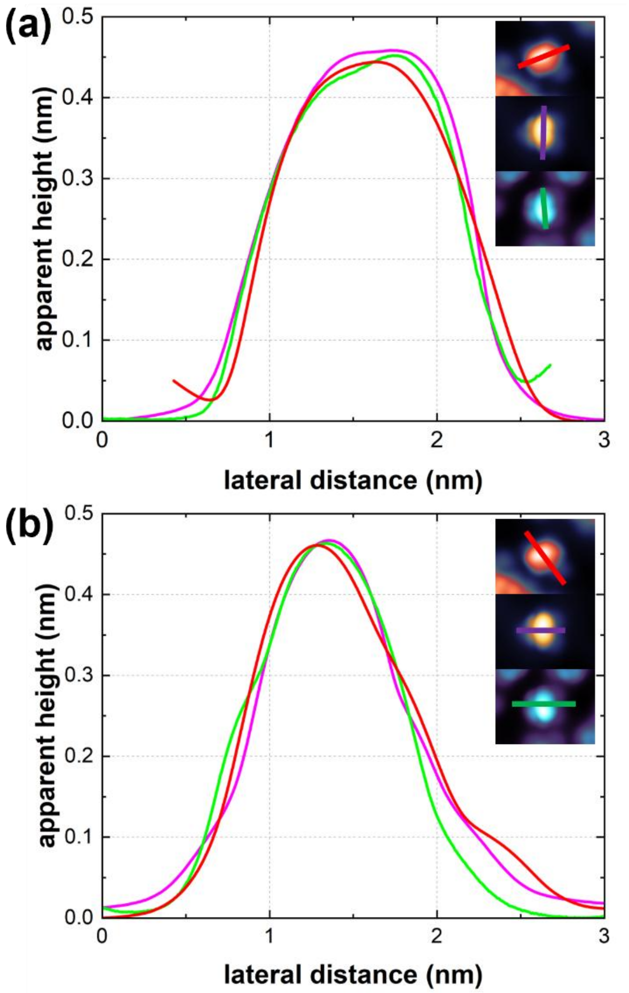

Figure S28. Linescan comparison between all three rotors SubPc-Azo, SubPc-MN and SubPc-MP: (a) linescan along long side of SubPc base; (b) linescan along short side of SubPc base (red = SubPc-Azo, purple = SubPc-MN, green = SubPc-MP.

Linescan comparison SubPc-Azo, SubPc-MN and SubPc-MP

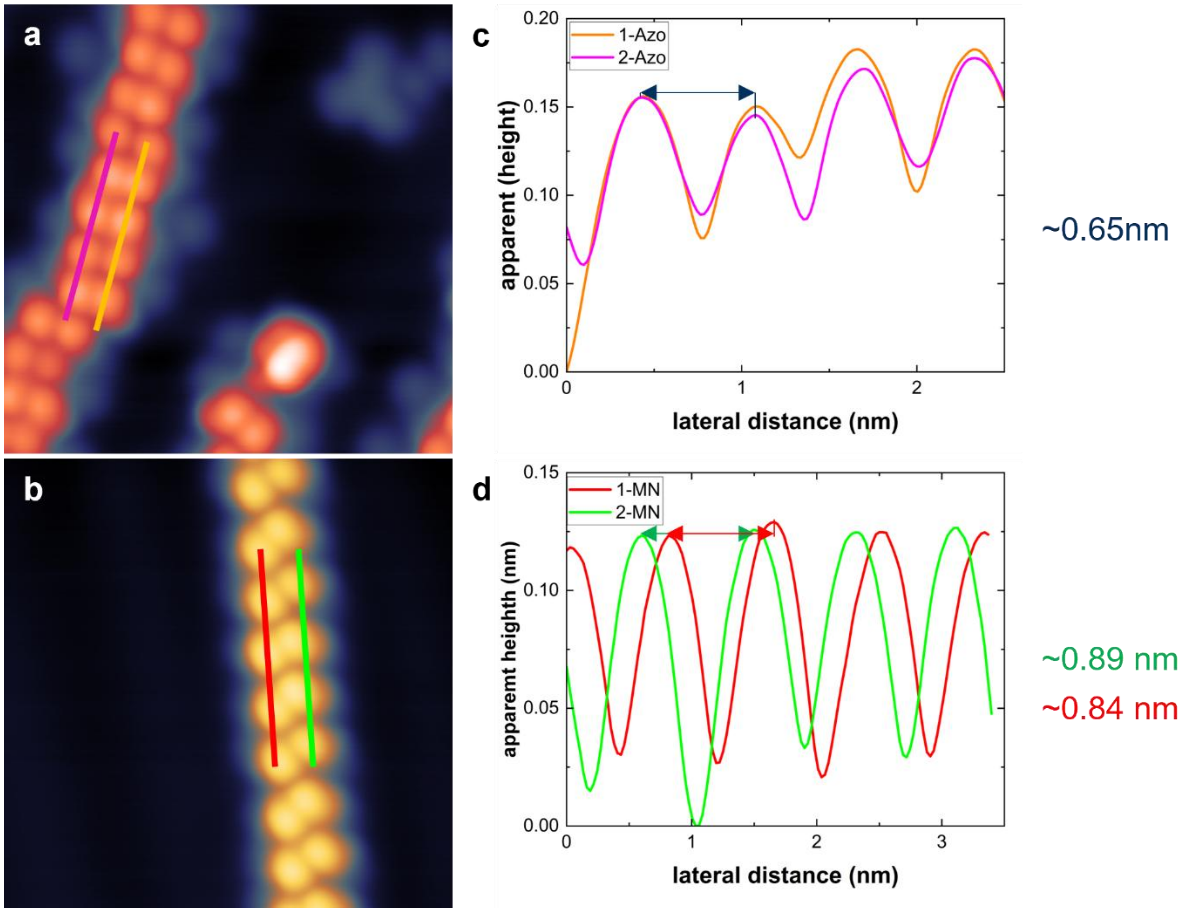

Figure S29. Linescan comparison between the chains of SubPc-Azo and SubPC-MN. (a) Chain of SubPc-Azo with two lobes in alternating stacking order; (b) SubPc-MN chain with two lobes in parallel order; (c) Corresponding linescans to (a), showing a parallel arrangement of the lobes with a distance of about 0.65 nm; (d) Corresponding linescans to (b), showing a shift of the lobes and a slightly larger separation of around 0.84 nm to 0.89 nm between the lobes along the chain.

Additional DFT calculations and simulated images

SubPc-MN DFT calculations and simulated images

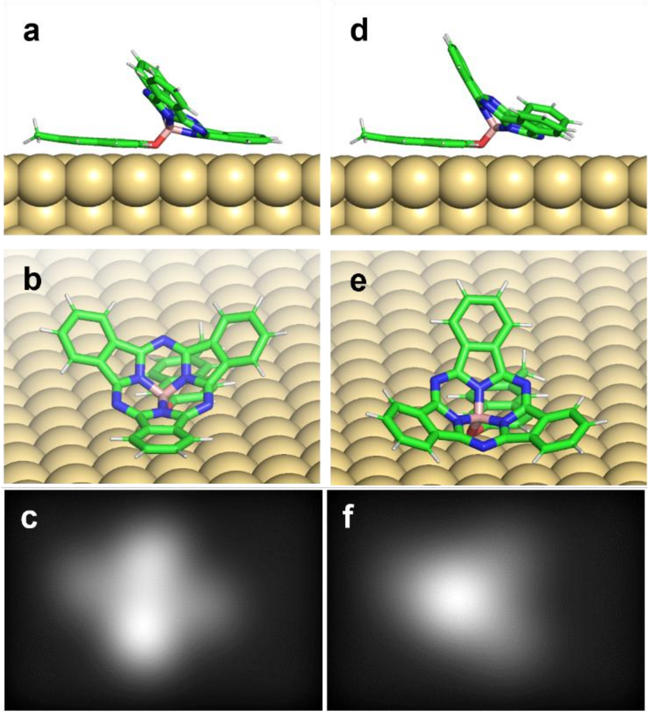

Figure S30. Reverse adsorption geometry of **SubPc-MN** calculated by DFT. (a) Conformation with two phenyl rings of the SubPc unit pointing out of the surface; (b) Corresponding view on the SubPc-base with two phenyl rings of the SubPc unit pointing out of the surface; (c) Corresponding simulated STM image with two phenyl rings of the SubPc unit pointing out of the surface (image size 3.0 nm x 4.0 nm); (d) Conformation with two phenyl rings adsorbed on the surface and the third pointing out; (e) Corresponding view on the SubPc-base with one phenyl ring of the SubPc unit pointing out of the surface; (f) Corresponding simulated STM image with one phenyl ring of the SubPc unit pointing out of the surface (image size 3.0 nm x 4.0 nm).

Comparison theoretical images of SubPC-chains to experimental images

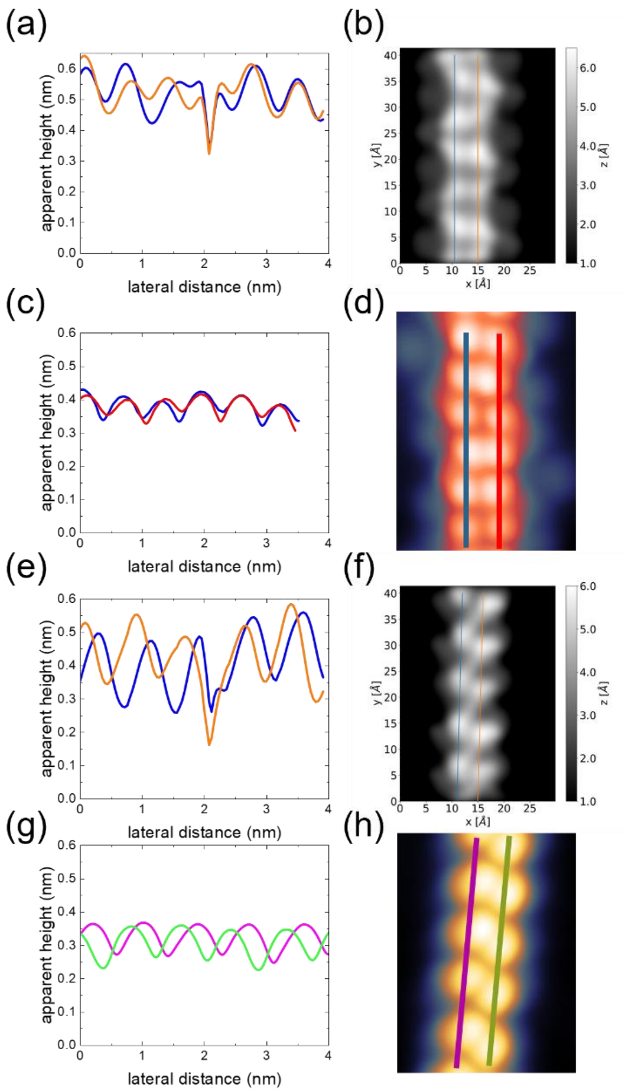

Figure S31. Linescan comparison between simulated images and experimental images for SubPc-Azo and SubPC-MN : (a) linescans on simulated SubPC-Azo chain; (b) simulated image of SubPc-Azo with lines; (c) linescans on experimental SubPC-Azo chain; (d) STM image of SubPc-Azo chain with lines (Image Parameters: : U = 0.5 V, I = 20 pA, 4 nm x 3 nm); (e) linescans on simulated SubPC-MN chain; (f) simulated image of SubPc-MN with lines; (h) STM image of SubPc-MN chain with lines (Image Parameters: : U = 0.2 V, I = 20 pA, 4 nm x 3 nm).

References

(1) Paton, A. S.; Lough, A. J.; Bender, T. P. One Well-Placed Methyl Group Increases the Solubility of Phenoxy Boronsubphthalocyanine Two Orders of Magnitude. *Ind. Eng. Chem. Res.* **2012**, *51* (18), 6290–6296. https://doi.org/10.1021/ie202998f.

(2) Shi, M.; Zhao, Y.; Xu, H.; Mack, J.; Yin, L.; Wang, X.; Shen, Z. Photoisomerization and Optical Properties of a Subphthalocyanine–Azobenzene–Subphthalocyanine Triad. *RSC Adv.* **2016**, *6* (75), 71199–71205. https://doi.org/10.1039/C6RA11452K.
