# Supplementary figures and images for: Subphthalocyanine Platform for Single-Molecule Machines on Surface: Ligand-Directed Adsorption on Au(111)
Source: ACS Nano. 2026 Mar 12;20(11):9139–46. doi: 10.1021/acsnano.5c17283 (PMC13019658; doi:10.1021/acsnano.5c17283)

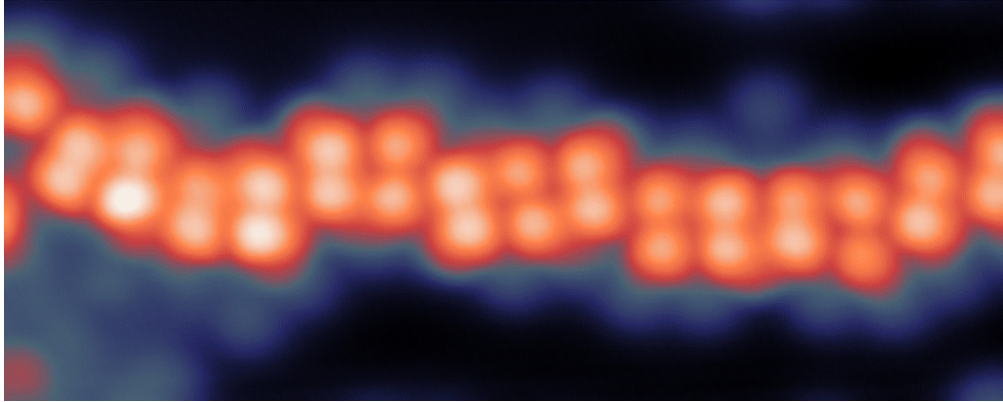

421x169mm (72 x 72 DPI)

Supplement: Supplementary file 2 [file nn5c17283_si_002.pdf]

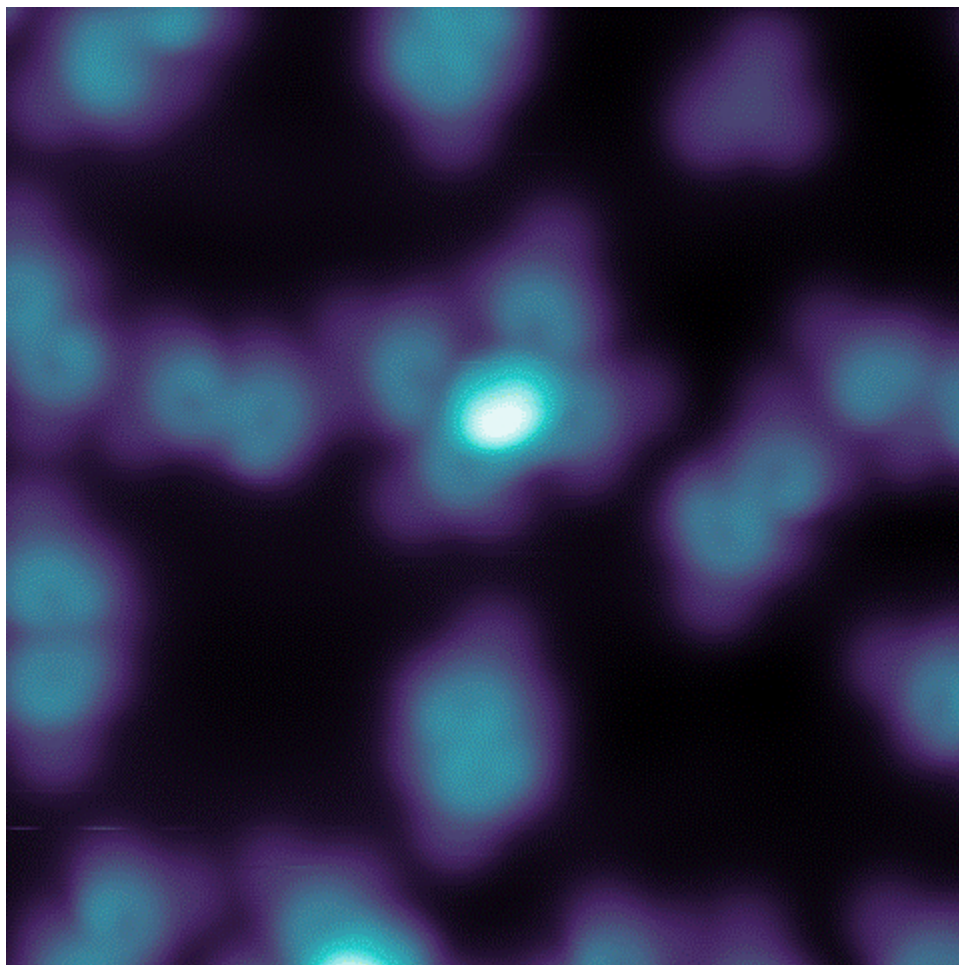

169x169mm (72 x 72 DPI)

Supplement: Supplementary file 8 [file nn5c17283_si_008.pdf]
